# Pac13 is a Small, Monomeric Dehydratase that Mediates the Formation of the 3′‐Deoxy Nucleoside of Pacidamycins

**DOI:** 10.1002/anie.201705639

**Published:** 2017-08-30

**Authors:** Freideriki Michailidou, Chun‐wa Chung, Murray J. B. Brown, Andrew F. Bent, James H. Naismith, William J. Leavens, Sean M. Lynn, Sunil V. Sharma, Rebecca J. M. Goss

**Affiliations:** ^1^ School of Chemistry University of St Andrews North Haugh St Andrews Fife KY16 9ST UK; ^2^ GSK Stevenage SG1 2NY UK

**Keywords:** dehydratases, enzymology, nucleosides, structural biology, UPA

## Abstract

The uridyl peptide antibiotics (UPAs), of which pacidamycin is a member, have a clinically unexploited mode of action and an unusual assembly. Perhaps the most striking feature of these molecules is the biosynthetically unique 3′‐deoxyuridine that they share. This moiety is generated by an unusual, small and monomeric dehydratase, Pac13, which catalyses the dehydration of uridine‐5′‐aldehyde. Here we report the structural characterisation of Pac13 with a series of ligands, and gain insight into the enzyme's mechanism demonstrating that H42 is critical to the enzyme's activity and that the reaction is likely to proceed via an E1cB mechanism. The resemblance of the 3′‐deoxy pacidamycin moiety with the synthetic anti‐retrovirals, presents a potential opportunity for the utilisation of Pac13 in the biocatalytic generation of antiviral compounds.

Nucleic acids play a central role in nature and modified nucleosides are present in a wide range of anti‐viral, anti‐cancer drugs and antibiotics.^1^ Though a variety of naturally occurring nucleic acid analogues exist, few include modifications to the ribose or deoxyribose ring. The uridyl peptide antibiotics (UPAs) pacidamycin, naspamycin, mureidomycin and sansanmycin, attract much attention^2^ with a clinically unexploited mode of action^1^ and an unusual biosynthetic assembly.^3^ Intriguingly, the (UPAs) contain a biosynthetically distinct 3′‐deoxyuridine that resembles the synthetic anti‐retrovirals such as stavudine **4**, abacavir **5** (Figure 1) and the cytotoxic natural product cordycepin **6**, the biosynthesis of which has not yet been determined.^4^ A detailed mechanistic understanding of the individual enzymes employed in the generation of the 3′‐deoxyuridine core is required in order to facilitate their future biotransformative potential.

**Figure 1:**
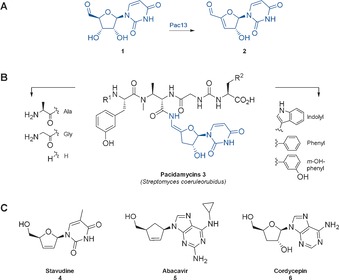
Medicinally relevant compounds containing 3′‐deoxy‐nucleoside moieties: A) biogenesis of the pacidamycin nucleoside motif: Pac13 catalyses dehydration of uridine‐5′‐aldehyde **1** to form 3′‐deoxy‐3′,4′‐didehydrouridine‐5′‐aldehyde **2**; B) pacidamycin, an uridyl peptide antibiotic (highlighted: the pacidamycin nucleoside core, the characteristic 3′‐deoxy uridine); C) stavudine **4** and abacavir **5**, widely used anti‐virals and cordycepin **6**, cytotoxic natural product.

In pacidamycin **3**, biosynthesis the 3′‐deoxy moiety is mediated by Pac13, an enzyme found to catalyze the key dehydration of uridine‐5′‐aldehyde **1** to form 3′‐deoxy‐3′,4′‐didehydrouridine‐5′‐aldehyde **2**. Previously proposed as a dehydratase^5^ investigations into the structure, kinetics and mechanism of this unusual enzyme remained to be performed. In stark contrast to most characterised dehydratases, Pac13 is small (121 aa), monomeric, co‐factor independent and utilises a non‐activated nucleoside, rather than a free monosaccharide, as a substrate.^6^ The biosynthetic uniqueness coupled to the potential synthetic utility inspired us to investigate Pac13’s structure and mechanism. This is the first mechanistic study of the formation of the 3′‐ deoxynucleosides in natural product biosynthesis. We demonstrate that not only is Pac13 unusually small, monomeric and cofactor independent, but that it is also mechanistically distinctive.

Dehydratases are important enzymes in primary and secondary metabolism and have been shown to mediate catalysis via a variety of mechanisms^7^ including metal‐dependent, acid‐base, radical^8^ and covalent9a mechanisms which we summarise in the Supporting Information (SI), Figure S15. All dehydratases studied so far that are involved in carbohydrate processing, to the best of our knowledge, have been shown to be large, multimeric and in general to require co‐factors (commonly metal ions or nucleotide) or needing covalent interactions for catalysis. A handful of dehydratases studied so far utilise a more unusual E1cb mechanism, these include the well‐studied dTDP‐d‐glucose 4,6‐dehydratase RmlB6a and the UDP‐Glc*N*Ac 5,6‐dehydratase TunA,6b both nicotinamide‐dependent (Figures S15 and S16, SI p. 21). RmlB and TunB mediate an NAD^+^ assisted oxidation of a hydroxy group within their nucleotide substrates, followed by the enzymes’ mediation of an E1cb dehydration to generate the respective enones, and a subsequent reduction of the resultant conjugated *C=C* double bond in the case of RmlB (Figure S16).6a 3‐dehydroquinate dehydratase I (DHQ I) is a dimeric enzyme which catalyses the third step of the shikimate pathway in a variety of organisms. It relies upon an essential Schiff base formation (Figure S16) between the substrate and a conserved lysine for catalysis that leads to an E1cb elmination.9b

Bioinformatic analysis of Pac13 using BLAST^10^ and HHpred,^11^ reinforced Pac13’s similarity to both metal dependent and metal independent cupins,^12^ including lyases and isomerases (Tables S5–S7). Heterologous expression, purification and crystallisation resulted in Pac13 wt crystals that diffracted to 1.55 Å. Solution of the structure required the preparation of the seleno‐methionine derivative (SeMet‐Pac13) and demonstrated that the enzyme was indeed a cupin (Figure 2). Pac13 is the only monomeric, metal‐free cupin dehydratase characterised to date (Table S8). Cupins generally occur as components of multimeric complexes and are frequently accompanied by metal ions,12b in contrast Pac13 is small, discrete and metal independent. Comparison of the Pac13 coordinates with the PDB archive using the Dali server^13^ (Table S6) revealed that the two closest homologues are cupins of non‐assigned function, while the third is the lyase KdgF^14^ (PDB: 5fpx, Z‐score 13.4, RMSD 2.1). KdgF is a dimeric, cupin (Figure S18) requiring a M^2+^ for the conversion of 4,5‐unsaturated galacturonate (Δ*G*alUA) to 5‐keto‐4‐deoxyuronate (DKI), a reaction distinct to Pac13’s chemistry.

**Figure 2:**
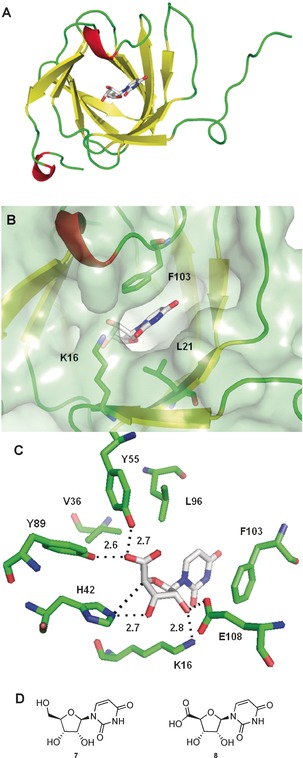
A) Crystal structure of Pac13 (PDB: 5OO5), refined as the monomer to 1.55 Å, MolProbity score of 96 %. Cartoon representation of Pac13 with uridine 5′‐uronic acid **8**. B) Active site “pocket**”** of Pac13 with uridine 5′‐uronic acid **8**. Residues K16, L21, F103 and the ligand are shown as stick representation. Pac13 is shown as cartoon and surface. C) Active site of Pac13 with uridine 5′‐uronic acid **8**. The active site residues Y89, Y55, K16, E108 and F103 and their distances from the ligand functional groups are shown. V36 and L96 are also shown. D) Compounds that were successfully used for crystal soaks: uridine **7**, uridine 5′‐uronic acid **8**.

Whilst the cupin fold^12^ is common for isomerases and epimerases (eg RmlC^[15])^ as shown in the CATH database (Figure S18) it is not common for lyases and ectoine synthase (EtcC) is the only other cupin shown function as a dehydratase, (Table S8, Figure S21). Perhaps such dehydratase activity will be seen to be very rare for cupins as the open β‐barrel does not assist in the protection of the product from water. EtcC is dimeric and Fe^2+^ dependent, catalysing the formation of ectoine by ring closure of the substrate *N*‐γ‐acetyl‐l‐2,4‐diaminobutyric acid.

The open active site of wt Pac13 did not allow us to locate the substrate binding site with confidence. Initial soaks with uridine‐5′‐aldehyde **1**, the natural substrate, were problematic due to both turnover and the inherent instability of this molecule. In order to overcome this problem we used substrate analogues, uridine **7** and uridine 5′‐uronic acid **8**, which enabled us to obtain liganded complexes of the enzyme, providing the first clues of the location and structural determinants of the active site (Figure 1). Both ligands occupied the same binding pocket in their respective co‐crystal structures (Figure S20). Comparison of the apo‐ and ligand‐bound structures revealed that small movements in residues such as K16, F103 and L21 create a pocket that encloses the ribose ring of the substrate analogues but leave one side of the uracil group solvent exposed (Figure 1). Within the ribose pocket, the 5′ oxygen lies almost equidistant (2.5–2.7 Å) between Y55 and Y89, with a slightly shorter distance to Y89, whereas the 4′ proton and 3′ hydroxy are 2.7–3 Å from H42.

This leaves the 2′ hydroxy of the ribose ring coordinated by the side chains of K16 and E108. In contrast to this extensive network of direct polar interactions the uracil ring makes a single H‐bond to K16 at the lip of the active site, with more Van der Waals (L21, V34, V99) and π‐stacking interactions (F103) characterising recognition of this part of the substrate. Notably, lipophilic residues L97 and V36 border the active site, creating a hydrophobic environment and perhaps sheltering the substrate or dehydrated product from attack from water. Pac13’s substrate‐binding site is distinct to the active site environment of the most well characterised cupin isomerases, such as RmlC^15^ (Scheme S1), suggesting a unique mechanism.

The observed electronic environment in the active site caused us to disfavour series of mechanisms. An E2 mechanism is unlikely; the substrate's stereochemistry prevents the required antiperiplanar relationship between the proton being abstracted and the exiting hydroxy. It is unlikely that an E1 mechanism (Scheme 1 B) operates, as there is no highly acidic residue proximal to the 3′ hydroxy group that could assist the stabilisation of a carbocation. A dehydroquinase type I mechanism (Figure S16) can be eliminated as the possibility of a covalent lysine linkage does not exist. Furthermore a radical mechanism (Scheme 1 C) could be excluded due to the absence of a metal ion or prosthetic group. An E1cB mechanism can be postulated from the structure to be likely (Scheme 1 A): in the first step, H42 could act as base extracting the activated 4′ proton, creating an enolate **10**. The oxyanion could then be stabilised by hydrogen bonding by Y55 and Y89, and the protonated H42 could then act as the acid, donating a proton to 3′ hydroxy group of the substrate and thus generating a better leaving group. Alternatively, as informed by the crystal structures of the Pac13 complex, E108 could play a dual role: substrate binding and acting as the active‐site acid, through a coordinated water molecule (Figure S19). Electrons moving from the enolate could lead to water elimination at 3′ position and generation of the desired product. Residues F103 and K16 could simply be involved in the orientation and binding of the substrate, rather than in catalysis. This proposed mechanism is perhaps closest to the E1cb mechanism postulated to be utilised by DHQ II, a dodecameric enzyme with a flavodoxin‐like fold9b (Scheme 2), but distinctive with Y89 and Y55 potentially acting to stabilise the enolate anion and H42 implicated as possibly playing the role the role of both acid and base.

**Scheme 1:**
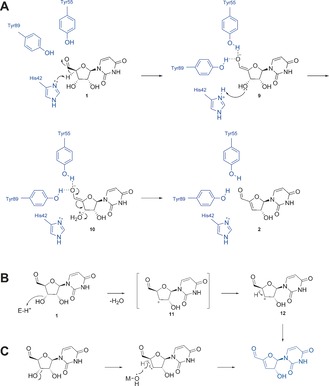
Proposed mechanism for Pac13‐mediated dehydration of uridine‐5′‐aldehyde **1** to to form 3′‐deoxy‐3′,4′‐didehydrouridine‐5′‐aldehyde **2**. Initially speculated Pac13‐mediated dehydratase mechanisms: A) E1cB mechanism; B) carbocation formation; C) radical formation.

**Scheme 2:**
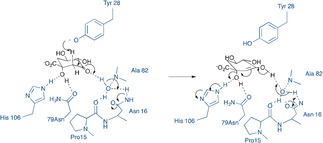
Proposed E1cb mechanism for DHQ II, depicting Tyr28 acting as base to catalyse enolate formation, with the enolate being stabilised by hydrogen bonding to regions oft he peptide backbone, via a bridging water molecule. Figure adapted from Ref. 9b.

To probe Pac13 function, the reaction of Pac13 with uridine‐5′‐aldehyde **1** was monitored by LC‐MS. A species with *m*/*z* 225, corresponding to the 3′‐deoxy‐3′,4′‐didehydrouridine‐5′‐aldehyde **2** was detected. No conversion could be seen in enzyme free controls. In aqueous conditions, **1** exists completely as its hydrate form.3c Next, we followed the reaction by ^1^H NMR and observed the disappearance of the 4′ proton multiplet at 4.0 ppm. The characteristic 3′ proton could be seen to shift downfield, appearing as a doublet of doublets at 5.4 ppm, consistent with generation of an olefinic bond, and enabling reaction monitoring. Unlike other dehydratase‐mediated reactions that exist in equilibrium,^7^ we demonstrate here that the Pac13‐catalysed dehydration of uridine‐5′‐aldehyde reaches completion. However, rapid abstraction of the 4′ proton in comparison to the depletion of other substrate protons such as H5 and H6, indicative of an E1Cb mechanism, was not observed (Figure S24‐5, SI p. 33). COSY and HSQC NMR of the enzymatic preparation allowed us to confirm the structure of the expected product. The optimal conditions for Pac13 activity were then examined. NADH, NADPH, MgCl_2_ were found not to be required. Based on extensive crystallographic and biochemical analysis, including enzymatic assay in presence of 100 mm EDTA, requirement of a metal for either catalysis or structural integrity of the protein could be confidently excluded. Kinetic constants were determined by following the course of the reaction by HPLC; these studies revealed a relatively low reaction rate with a high *K*_M_ (*k*_cat_=3.63±0.3 min^−1^, *K*_M_=5.86±0.9 mm). Whilst this slow rate is consistent with those reported for other) enzymes involved in secondary metabolism,^16^ it may also reflect the low concentration of aldehyde present as the substrate uridine‐5′‐aldehyde **1** exists in equilibrium with its hydrate form in the aqueous condition of the enzymatic assay. In more native conditions, the *K*_M_ may be significantly lower as it is postulated that the aldehyde substrate of Pac13 could be channelled from the previous biosynthetic enzyme and protected from bulk solvent.

A detailed pH profile analysis demonstrated that the enzyme was completely inactive below pH 5.4 and above pH 10. The profile followed a bell‐shaped curve (Figure S13B), with p*K*a_1_ and p*K*a_2_ values of 6.2 and 8.6, respectively. Since the substrate does not have any ionisable groups in this pH range, this result supports our proposal of a histidine acting as a general base to abstract the 4′ proton.^17^ There is no obvious residue positioned to protonate the leaving hydroxide ion and this role may be fulfilled by H42 or by a solvent molecule mediated through E108 to eventual His deprotonation. Though unusual, a histidine residue mediating as a general base and acid in two sequential steps, has been previously reported (Figure S16 and SI p. 33).^18^

To gain further confidence with regards to our proposed mechanism, we carried out a series of site‐directed mutagenesis (SDM) studies. Mutants E108Q, K16R, H42Q, Y55F and Y89F were produced and purified, affording diffracting crystals of the same morphology and space group as wt Pac13, which raised our confidence that the mutants folded correctly and that the effects seen are specific to interactions with each mutated residue rather than global effects on protein folding and structure.

The mutation of H42 to a Q completely abolished activity, further supporting our hypothesis that H42 acts as a base for the abstraction of the 4′ proton and possibly for the protonation of the 3′ hydroxy group. The kinetic measurements for the activity of Y55F and K16R demonstrated lower affinity and catalytic efficiency than wt Pac13 (Table 1). Mutation of K16 to an R did not abolish activity, further demonstrating that K16 is not an essential lysine involved in a Schiff‐base formation; hence a DHQ I‐type mechanism could be further excluded with confidence. Y89F demonstrated a significant loss in activity compared to the wt (Table 1). Mutation of the E108 resulted in almost no activity; a result that could perhaps be attributed to the importance of E108 both in stabilizing substrate binding and having a role in catalysis. Indeed, given the open active site of Pac13, as observed in the crystal structure, the existence of a residue that would maintain the substrate in a productive orientation appears to be essential. Finally, the significant loss of activity with mutants Y89F and Y55F is consistent with their proposed involvement in stabilizing the oxyanion and underlines their important role for catalysis.

**Table 1:** Kinetic constants for Pac13 WT and mutants.

Enzyme	*K*_M_ [mm]	*k*_cat_ [min^−1^]	*k*_cat_/*K*_M_ [min^−1^ mm ^−1^]	% conversion at 1 mm [S]
WT	5.86±0.9	3.63±0.3	0.62	43
K16R	6.40±1.3	0.79±0.1	0.12	24
Y55F	8.13±1.2	0.71±0.1	0.09	19
Y89F	n.d.^[a]^	n.d	n.d	10
E108Q	n.d	n.d	n.d	4
H42Q	n.d	n.d	n.d	0

[a] n.d.=not determined.

The biochemical data were in accordance with the data obtained from structural analysis of the mutants. In the H42Q–uridine complex (Figure 3 A,C), the critical interactions between 5′ OH and Y55 and Y89 remained the same as in the WT‐uridine structure, additionally the hydrogen bond between K16 and E108 with 2′‐OH, as well as the π‐stacking of the uracil ring with F103 are all preserved. Q42 appears to have the same distance from the 4′ proton and the overall fold of the protein does not seem to be impacted, all of which suggests that the H42Q mutation affects the key catalytic step and not substrate binding. Similarly, on examining the Y89F–uridine complex (Figure 3 B,D) and Y55F–uridine complex structures (Figure S23, Table S4), we observed a high degree of similarity with the wt enzyme. Although there are subtle shifts in the position of both the ribose and uracil rings, of the substrate, and in the orientation of the 5′ functionality, these changes are small and consistent with our hypothesis that decreased catalytic efficiency of these mutants is largely due to inability of the phenyl mutants to stabilize the transient 5′ oxo‐anion that forms during catalysis, rather than having a major effect on binding competency.

**Figure 3:**
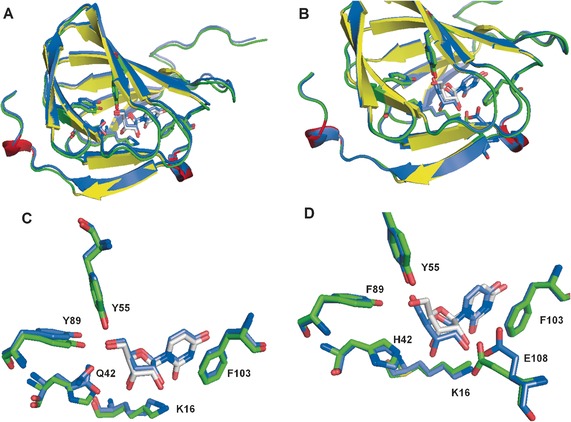
A, C) Complex of H42Q with uridine (colored in purple, PDB: 5OO8) superimposed with Pac13 wt complex with uridine (colored in yellow and gree, PDB: 5OO4). B, D) Complex of Y89F with uridine (colored in purple, PDB:5OOA) superimposed with Pac13 wt complex with uridine (colored in yellow and green).

This study constitutes the first structural and mechanistic investigation into the biosynthesis of the intriguing 3′‐deoxy nucleoside moiety of the UPAs. Through a series of crystallographic and biochemical experiments, supported by SDM and kinetics, we provide insight into the mechanism of the unique C=C bond formation orchestrated by Pac13. We demonstrate here that the reaction most likely proceeds via an E1Cb mechanism, with H42 acting as an active site base, however, unlike many other dehydratases, no metal or cofactor is required for activity. Our deepened understanding of this biosynthesis paves the way toward a biosynthetic alternative to highly valuable yet, synthetically challenging 3′‐modified nucleosides. Our next goal is to further explore and challenge Pac13 so as to develop a promising biocatalyst.

## Conflict of interest

The authors declare no conflict of interest.

## Supporting information

As a service to our authors and readers, this journal provides supporting information supplied by the authors. Such materials are peer reviewed and may be re‐organized for online delivery, but are not copy‐edited or typeset. Technical support issues arising from supporting information (other than missing files) should be addressed to the authors.

Supplementary
