# Supplementary material for: Pac13 is a Small, Monomeric Dehydratase that Mediates the Formation of the 3′‐Deoxy Nucleoside of Pacidamycins
Source: Angew Chem Int Ed Engl. 2017 Aug 30;56(41):12492–7. doi: 10.1002/anie.201705639 (PMC5656905; doi:10.1002/anie.201705639)
Supplement: Supplementary file 1 — Supplementary [file ANIE-56-12492-s001.pdf]

Angewandte Chemie
Eine Zeitschrift der Gesellschaft Deutscher Chemiker

Supporting Information

Pac13 is a Small, Monomeric Dehydratase that Mediates the Formation of the 3′-Deoxy Nucleoside of Pacidamycins

*Freideriki Michailidou, Chun-wa Chung, Murray J. B. Brown, Andrew F. Bent, James H. Naismith, William J. Leavens, Sean M. Lynn, Sunil V. Sharma, and Rebecca J. M. Goss**

anie_201705639_sm_miscellaneous_information.pdf

Table of Contents

Experimental Procedures 2
General Experimental 2
Generation of *pac13* expression vectors 3
Preparation of the *pac13/*pSG181 expression vector 3
Site directed mutagenesis 3
Protein production and purification 4
Pac13 and SeMet Pac13 production and purification 4
Production and purification of Pac13-His_8 wt and mutants 6
Protein crystallisation 7
Data collection, processing and refinement 8
Synthesis of uridine-5′-aldehyde, 1. 12
Uridine-5′-uronic acid. 16
NMR assay of Pac13 with uridine-5′-aldehyde 1 16
HPLC assays, steady-state kinetic and pH profile analysis 17
LC-MS analysis of enzymatic assays 19
HPLC purification of uridine-5′-aldehyde 19
Results and Discussion 20
Dehydratases: an overview of the main classes 20
Bioinformatic and structural analysis of Pac13 22
Aspects of the chemistry of cupins 26
Site Directed Mutagenesis 29
pH profile analysis of Pac13 and mechanistic information derived from NMR assays at two different pH values 30
References 31

Experimental Procedures

General Experimental

All starting materials and reagents were commercially available and were used without further purification, unless otherwise stated. NMR spectra were acquired on either Bruker Avance 300 (^{1}H at 300 MHz, ^{13}C at 75 MHz), Bruker Avance II 400 spectrometer (^{1}H at 400 MHz, ^{13}C at 100 MHz), Bruker Avance 500 spectrometer or Bruker Avance III 500 (^{1}H at 500 MHz, ^{13}C 126 MHz), Bruker Avance II 600 (^{1}H at 600 MHz, ^{13}C 150 MHz). Chemical shifts δ are reported in parts per million (ppm) and are quoted relative to centre of the reference non-deuterated solvent peak for δ_H (CDCl3: 7.26 ppm; CD_3OD: 4.87, 3.31 ppm, D_2O: 4.79 ppm) and δ_C ($CDCl_3$: 77.16 ppm; CD_3OD: 49.00 ppm). ^{13}C NMR spectra were recorded with ^{1}H decoupling, while all spectra were processed and analysed using MestReNova 8.0.1. Coupling constants *J* are given in Hertz (Hz). Multiplicity patterns are described as:, s - singlet, t - triplet, d – doublet, dd - doublet of doublets, , apt – apparent triplet. m - multiplet. ^{1}H, COSY, HSQC and HMBC experiments were carried out as required during the process of structural assignment for compounds. HRMS measurements were recorded by the EPSRC UK National Mass Spectrometry Facility at Swansea University. Milli-Q deionized water was used in the preparation of all buffers. Analytical HPLC was performed using an Agilent 1260 Infinity instrument. LC-MS measurements were recorded using a Thermo Velos Pro / Orbitrap

Velos Pro equipped with H-ESI source and Thermo Scientific Dionex UltiMate 3000 RS chromatography system. Preparative HPLC was performed using a Waters 515 HPLC instrument, equipped with a Waters 2296 photodiode array detector and a Waters 2767 sample manager. Plotting and analysis of the kinetic data was performed by Origin or GraphPad.

The pET-28a(+) and pET-21a(+) plasmids were used for gene cloning and were obtained from Novagen, Merck Biosciences, while the pSG181 plasmid has been previously prepared by Goss group members. *S. coeruleorubidus* AB 1183F-64, the wild type pacidamycin producer, was provided by ATCC Manassas (USA), and was used as a spore stock. *E. coli* DH10B-T1 cells were provided by John Innes Centre. E. coli BL21 (DE3) and *E. coli* BL21 Codon Plus RIPL (DE3) were purchased by Novagen, Merck Biosciences.. Synthetic oligonucleotides for SDM were purchased by IDT (Integrated DNA Technologies, Belgium) and for PCR amplification of the *pac13* gene from ThermoScientific (UK). DNA sequencing was performed by GATC Biotech (Germany) and then GlaxoSmithKline (in-house service). IMAC purification and dialysis procedures were performed at 4 °C, whereas size exclusion chromatography was performed at ambient temperature. Protein concentration was measured by A280 absorbance (Nanodrop). Protein purity was assessed by SDS-PAGE (10 % polyacrylamide gel made in-house or Novex™ 4-20% Tris-Glycine, Invitrogen, UK) and intact LC-MS was used for protein identification (GlaxoSmithKline or University of St Andrews). Protein MW was calculated by Expasy ProtParam tool (ref). His_8-tagged proteins were utilized without further modifications, unless otherwise stated. Incubation was performed using a New Brunswick Scientific I26R Incubator / Shaker and a New Brunswick Scientific innova 4300 Incubator / Shaker. Centrifugation was performed using a Beckman Avanti J-25 centrifuge fitted with JLA-8.1000 and JA-12 rotors, a Thermo Scientific IEC CL3OR centrifuge and a Thermo Scientific accuspin microcentrifuge.

Proteins used for crystallization trials were thawed on ice and centrifuged (13000 rpm, 10 min, 4 °C) beforehand, while all buffers and crystallisation screens were kept at 4 °C and compound stocks at -20 °C or -80 °C. Buffers, crystallization screens (PACT, SG1) and crystallisation plates were purchased from Molecular Dimensions (MRC). Crystallization screens were performed by the sitting drop method, using a Mosquito HTS instrument (TTP labtech), the plates were incubated in Rockimager RI 1000 (Formulatrix), with the temperature controlled at 20 °C. Crystallisation using the hanging drop method, was performed manually using 24 or 15-well plates and the latter were incubated in an incubator with a temperature controlled at 20 °C.

Generation of *pac13* expression vectors

Preparation of the *pac13*/pSG181 expression vector

The *pac13* gene was PCR amplified from *g*DNA extracted from *S. coeruleorubidus* AB1183F-64 using the forward 5′-AAGCTTAGTAAGGGCTCTCGCTTTCACTG-3′ and 5′-CCCAAGCTTAGTAAGGGCTCTCGCTTTCACTG-3′ as the reverse primer. The purified PCR product was digested (NdeI, HindIII) and ligated into linearised pET-28a(+) (Novagen) following the standard protocol and confirmed by DNA sequencing. The *pac13*/pSG181 plasmid was constructed by restriction digestion of the parent plasmid *pac13*/pET-28(a) (NdeI, HindIII) and re-insertion into digested and linearised pSG181. The identity of *pac13*/pSG181 vector was verified by sequencing at GATC (Germany).

Site directed mutagenesis

The following point mutations were constructed by PCR amplification using *pac13*/pSG181 plasmid (5 ng/µl) as a template. Each reaction contained DMSO (2.5 µl), reverse and forward primer (2 µl of each 100 µM stock), DNA template (2 µl), 2X Phusion polymerase master mix with HF buffer (25 µl, ThermoScientific), sterile water (14.5 µl). The PCR protocol consisted of an initial step of 98 °C for 3 min, followed by 16 cycles of 98 °C for 1 min, 60 °C for 30 sec, 72 °C for 5 min and then a final step of 72 °C for 10 min. The template DNA was digested with DpnI (2 µl, 20000 U/ml, NewEngland Biolabs) for 1 h at 37 °C and transformed into TOP 10 competent cells. The sequence was verified by DNA sequencing performed in house (GlaxoSmithKline).

Mutant	Primer	Sequence
**Pac13 H42E**	Forward 5′-3′	GAGTGTCGAGCGCGGGGAGTTTCAGGAATTCTTC
	Reverse 5′-3′	GAAGAATTCCTGAAACTCCCCGCGCTCGACACTC
**Pac13 H42Q**	Forward 5′-3′	CGAGCGCGGGCAGTTTCAGGAATTC
	Reverse 5′-3′	GAATTCCTGAAACTGCCCGCGCTCG
**Pac13 E108Q**	Forward 5′-3′	CATTCAATGAACAAGATCAACGGCATGTCAGGTTC
	Reverse 5′-3′	GAACCTGACATGCCGTTGATCTTGTTCATTGAATG
**Pac13 K16R**	Forward 5′-3′	GGAAAGATTCAACAGGCATGGCATCGACC
	Reverse 5′-3′	GGTCGATGCCATGCCTGTTGAATCTTTCC
**Pac13 Y55F**	Forward 5′-3′	GAGTTCCTATACCTTTTACGTCGTCAGCGG
	Reverse 5′-3′	CCGCTGACGACGTAAAAGGTATAGGAACTC
**Pac13 Y89F**	Forward 5′-3′	CACCCGAATACACTTCTTCGGGTCGATG
	Reverse 5′-3′	CATCGACCCGAAGAAGTGTATTCGGGTG

Protein production and purification

Pac13 and SeMet Pac13 production and purification

The *pac13*/pSG181 plasmid was transformed in *E. coli* BL21 RIPL (DE3) chemically competent cells. *Growth conditions for wt Pac13;* A single colony obtained from a fresh transformation was inoculated in ZYP-0.82 (prepared in-house), supplemented with 50 µg/ml kanamycin and was incubated at 200 rpm and 37 °C for 8 h. Following inoculation of Super Broth based Auto Induction media (Formedium, UK) containing 50 µg/ml and kanamycin was left to incubate at 16 °C, at 200 rpm for 48 h. The cells were harvested by centrifugation (4000 rpm, 4 °C, 30 min) and the pellets were washed with cold lysis buffer and stored at -80 °C prior to purification. *Growth conditions for SeMet Pac13;* A single colony obtained by a fresh transformation was inoculated in 500 ml Lysogeny Broth (prepared in-house), supplemented with 50 µg/ml kanamycin and was incubated overnight at 200 rpm and 37 °C. The cells were harvested by centrifugation (3000 rpm, 25 °C, 20 min) and resuspended in minimal media (X 3). The Glucose free nutrient mix (Molecular Dimensions, UK) was dissolved in minimal media (1.1 g of nutrient mix/50 ml of media) and was filtered under sterile conditions. 950 ml of minimal media containing 50µg/ml kanamycin, 50 g glycerol and 50 ml of the prepared nutrient mix solution, were inoculated with 20 ml of the resuspended cells. After incubation at 37 °C, at 200 rpm for 15 min, followed by addition of 60 mg of selenium methionine (Sigma Aldrich, UK) the culture was left to reach an OD_{600} of 0.6. After addition of 100 mg/L lysine, 100 mg/L phenylalanine, 100 mg/L threonine, 50 mg/L isoleucine and 50 mg/L valine, and incubation for 20 additional min, gene expression was induced by 1 mM IPTG. The temperature was dropped to 20 °C and the culture was incubated for 24 h at 200 rpm. The cells were harvested by centrifugation (4000 rpm, 4 °C for 30 min) and the pellets were washed with cold lysis buffer (50 mM NaH_2PO_4, 300 mM NaCl, 20 mM imidazole, pH 7.8) and stored at -80 °C prior to purification.

Protein purification. The recombinant proteins bearing an *N*-terminal fusion His_8 were purified using immobilised affinity chromatography (IMAC). The cell pellets were resuspended in cold lysis buffer (50 mM NaH_2PO_4, 300 mM NaCl, 20 mM imidazole, pH 7.8, ~1 g of cell pellet/10 ml of lysis buffer) and lysed by cell disruption through a French press at 30 kpsi and 4 °C, (Constant Systems Ltd) two cycles of lysis. The supernatant was obtained by centrifugation at 16000 rpm, 4 °C for 30 min and was then combined with Ni-NTA agarose (Invitrogen, UK), pre-equilibrated with binding buffer (50 mM NaH_2PO_4, 300 mM NaCl, 20 mM imidazole, pH 7.8). The supernatant-resin suspension was gently agitated for 1 h and was loaded into a gravity flow column (Biorad, UK). Non-related proteins were removed by employing an imidazole gradient (50 mM NaH_2PO_4, 300 mM NaCl, pH 7.8, 20 mM and 40 mM imidazole). The recombinant protein was eluted (50 mM NaH_2PO_4, 300 mM NaCl, 300 mM imidazole, pH 7.8), combined with TEV protease (produced and purified in house), dialysed overnight against 100 mM NaCl, 50 mM NaH_2PO_4, pH 7.8 using dialysis tubing (2 K MWCO, Sigma Aldrich, UK) and then combined with Ni-NTA agarose (Invitrogen, UK), pre-equilibrated with the dialysis buffer (50 mM NaH_2PO_4, 100 mM NaCl pH 7.8). The proteins-resin suspension was gently agitated for 1 h and was loaded into a gravity flow column (Biorad, UK). The flow-through (containing the protein of interest) was collected and TEV protease was removed by employing an imidazole gradient (50 mM NaH_2PO_4, 300 mM NaCl, pH 7.8, 20 mM, 40 mM and 300 mM imidazole). The fractions were assessed by SDS-PAGE (10% polyacrylamide gel, made in-house), and the ones containing the protein of interest (wt protein without tag) were concentrated using Amicon Ultra Centrifugal Filter Units (3 K MWCO, Merck Millipore). A HiLoad 16/600 Superdex 75 prep grade column was equilibrated with size exclusion chromatography buffer (SEC, 50 mM Tris, 200 mM NaCl, pH 7.9) and loaded with 2.5 mL of the concentrated protein fraction from the the previous purification step. Pac13 was eluted with SEC buffer, collecting 1 mL fractions and the ones that contained pure Pac13 were dialysed against storage buffer (25 mM Tris, 100 mM NaCl, pH 7.9) and concentrated using an Amicon Ultra Centrifugal Filter Units (3 K MWCO, Merck Millipore). The purity was assessed by SDS-PAGE and the incorporation of Se atoms by LC-MS.

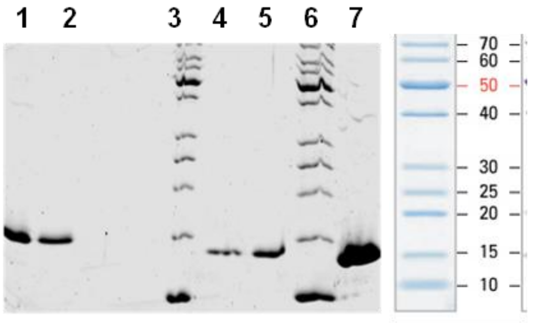

Figure S1. SDS-PAGE gel showing Pac13 purification process. (Lanes 3,6: Thermo Scientific PageRuler Unstained 200 kDa, lane 7: Pac13-His8 after Ni-NTA, lane 2: mixture of Pac13 and TEV protease after cleavage of the tag, lane 1: Pac13 after Ni-NTA following the cleavage step, lane 4: washing step of Ni-NTA, lanes 6,7: Pac13 after SEC).

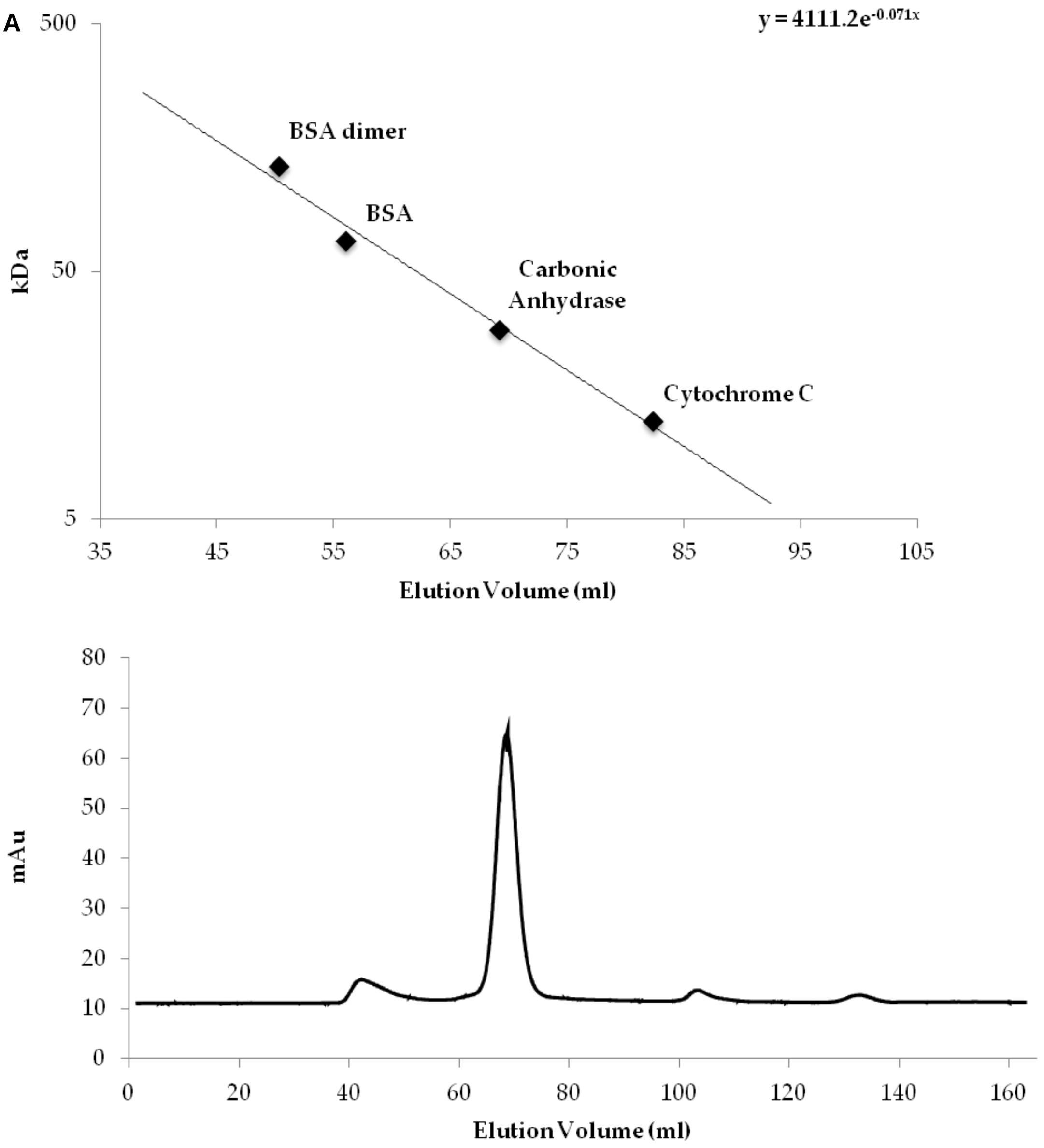

Figure S2. A Elution time of MW standards on SEC column HiLoad 16/600 Superdex 75 prep grade column. **B** UV trace of Pac13 SEC purification process.

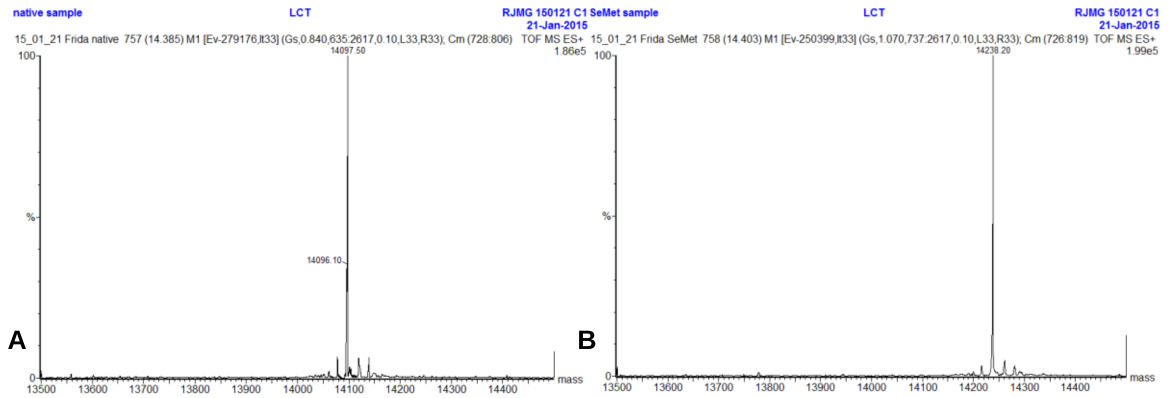

Figure S3. **A** MS of Pac13 – native sample. **B** MS of Pac13 – SeMet sample, showing m/z 14238.20, consistent with the incorporation of three Se atoms.

Production and purification of Pac13-His$_8$ wt and mutants

Plasmids were transformed in *E. coli* BL21 (DE3) chemically competent cells. A single colony obtained from a fresh transformation was inoculated in Lysogeny Broth (prepared in-house), supplemented with 50 µg/ml kanamycin and was incubated overnight at 200 rpm and 37 °C. 1 ml of the prepared starting culture was inoculated in 500 ml Overnight Express (Novagen) containing 50 µg/ml kanamycin and was left to incubate at 16 °C, at 200 rpm for 48 h. The cells were harvested by centrifugation (4000 rpm, 4 °C for 30 min) and the pellets were washed with cold PBS buffer and stored at -80 °C prior to purification. The recombinant proteins bearing an *N*-terminal fusion His$_8$ were purified using immobilised affinity chromatography (IMAC). The cell pellets were resuspended in cold lysis buffer (50 mM NaH_2PO_4, 300 mM NaCl, 20 mM imidazole, pH 7.8, ~1 g of cell pellet/10 ml of lysis buffer) and lysed by sonication on ice using a 19 mm probe (50 % amplitude, 7 min, 30 sec on, 10 sec off, 4 °C), two cycles of lysis. The supernatant was obtained by centrifugation at 16000 rpm, 4 °C for 30 min and was then combined with Ni-NTA agarose (Invitrogen, UK), pre-equilibrated with binding buffer (50 mM NaH_2PO_4, 300 mM NaCl, 20 mM imidazole, pH 7.8). The supernatant-resin suspension was gently agitated for 1 h and was loaded into a gravity flow column (Biorad, UK). Non-related proteins were removed by employing an imidazole gradient (50 mM NaH_2PO_4, 300 mM NaCl, pH 7.8, 20 mM and 40 mM imidazole). The recombinant protein was eluted (50 mM NaH_2PO_4, 300 mM NaCl, 300 mM imidazole, pH 7.8), buffer-exchanged into 25 mM Tris, 100 mM NaCl, pH 7.9 using Slide-A-Lyzer™ Dialysis Cassettes (3.5 K MWCO, 3-12 ml, ThermoScientific, UK) and concentrated using Amicon Ultra Centrifugal Filter Units (3 K MWCO, Merck Millipore). Protein concentration was measured by A280 absorbance (Nanodrop). Protein purity was assessed by SDS-PAGE (Novex™ 4-20% Tris-Glycine, Invitrogen, UK) and LC-MS was used for protein identification (GlaxoSmithKline).

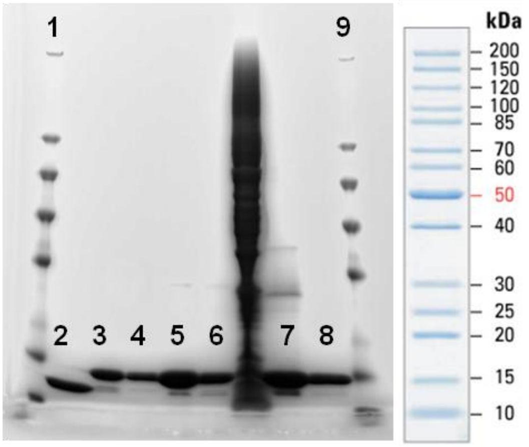

Figure S4. SDS-PAGE gel showing purified Pac13 wt and mutants. (Lanes 1,9: Thermo Scientific PageRuler Unstained 200 kDa, lane 2: Pac13, lane 3: Pac13-His$_8$, lane 4: K16R-His$_8$, lane 5: H42Q-His$_8$, lane 6: E108Q-His$_8$, lane 7: Y55F-His$_8$, lane 8: Y89F-His$_8$).

Table S1. Calculated protein molecular weight of Pac13-His$_8$ wt and mutants.

Protein	MW (Da)
wt Pac13	14096.67
wt Pac13-His$_8$	16364.00
E108Q Pac13-His$_8$	16363.01
H42Q Pac13-His$_8$	16354.98
K16R Pac13-His$_8$	16392.01
Y55F Pac13-His$_8$	16348.00
Y89F Pac13-His$_8$	16348.00

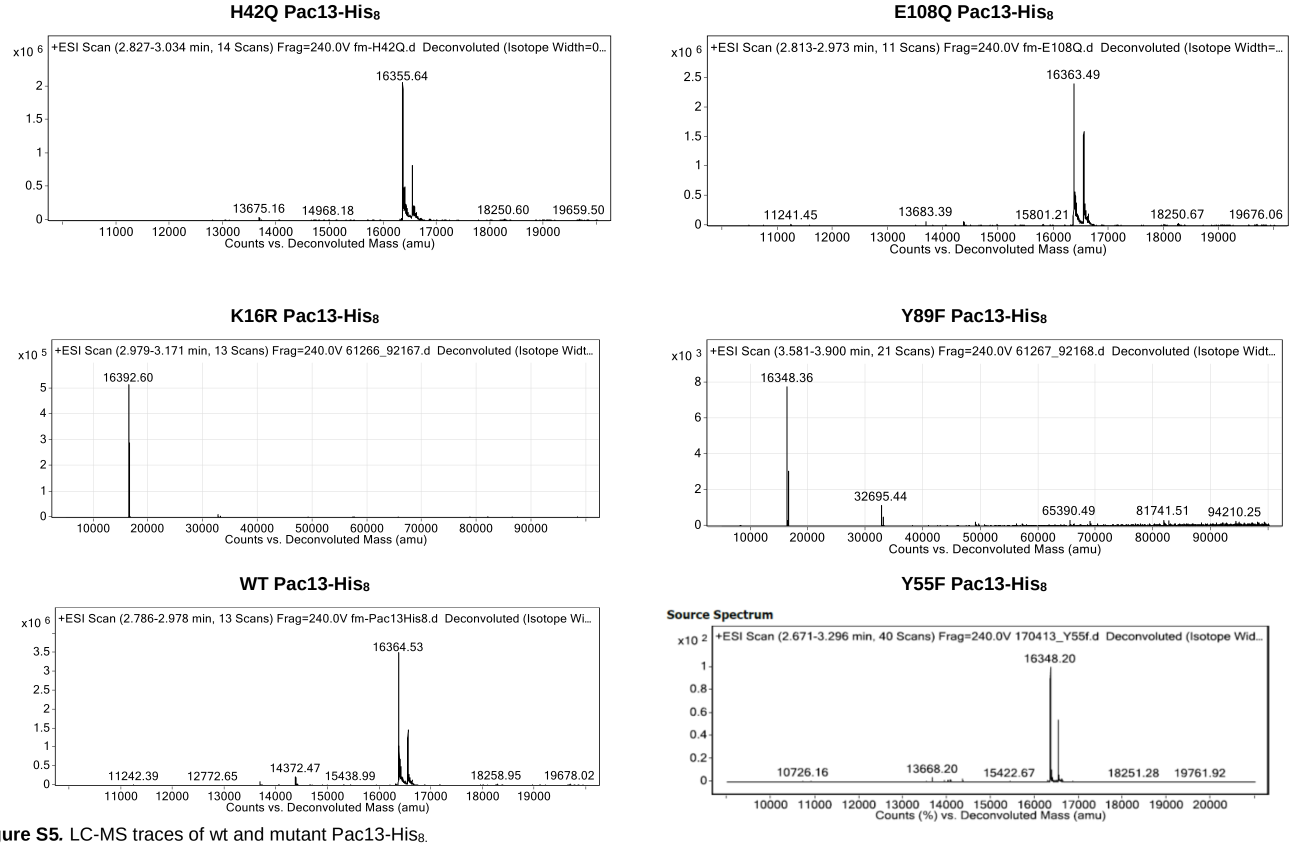

Figure S5. LC-MS traces of wt and mutant Pac13-His$_8$.

Protein crystallisation

Crystallisation of Pac13 wt and Pac13 SelMet.

Pac13 from *Streptomyces coeruleorubidus* at 1.8 mg/ml in 0.025 M Tris-base [tris(hydroxymethyl)aminomethane] pH 7.9, 0.1 M NaCl was used for crystallisation. Crystallisation was achieved employing the hanging drop method, using drops containing 2 µl protein, 1 µl well solution (0.1 M Tris [tris(hydroxymethyl)aminomethane] pH 8.8, 20% w/v poly[ethylene glycol] [PEG]-8K, 0.2 M $MgCl_2$) and wells of 500 µl. The 24-well plate was incubated in a 20 ºC incubator and crystals grew over a period of 3 days and were harvested into mother liquor supplemented with 20% glycerol before flash freezing in liquid nitrogen. Selenium-methionine (SeMet) crystals were obtained and handled using the same procedure.

Crystallisation of Pac13 wt and soaking with uridine and uridine 5'-uronic acid.

Pac13 from *Streptomyces coeruleorubidus* in 0.025 M Tris-base [tris(hydroxymethyl) aminomethane] pH 7.9, 0.1 M NaCl was used for crystallisation.

Soaking with uridine 5′-uronic acid. Crystallisation was achieved employing the hanging drop method, using drops containing 2 µl protein (1.6 mg/ml), 1 µl well solution (0.1 M Tris [tris(hydroxymethyl)aminomethane] pH 8.8, 16% w/v poly[ethylene glycol] [PEG]-8K, 0.2 M $MgCl_2$) and wells of 500 µl. The 24-well plate was incubated in a 20 ºC incubator and crystals grew over a period of 8 days. A single crystal was used for soaking in a 2 µl drop, containing mother liquor supplemented with uridine 5′-uronic acid **8** (stock in 0.1 M HEPES [4-(2-hydroxyethyl)-1-piperazineethanesulfonic acid] pH 7.4), in a final concentration of 0.02 M. Following incubation for 30 min at a 20 ºC incubator, the crystal was cryo-protected in mother liquor containing 20% glycerol, 0.001 M uridine 5′-uronic acid **8** and was flash-cooled in liquid nitrogen.

Soaking with uridine. Crystallisation was achieved employing the hanging drop method, using drops containing 1 µl protein (1.8 mg/ml), 1 µl well solution (90 mM Bis-Tris [2,2-Bis(hydroxymethyl)-2,2',2''-nitrilotriethanol] pH 6.5, 15% w/v poly[ethylene glycol] [PEG]-3350, 0.2 M Sodium acetate) and wells of 500 µl. The 24-well plate was incubated in a 20 ºC incubator and crystals grew over a period of 25 days. A single crystal was used for soaking in a 2 µl drop, containing mother liquor supplemented with uridine **7** (stock in MQ water), in a final concentration of 0.05 M. Following incubation overnight at a 20 ºC incubator, the crystal was cryo-protected in mother liquor containing 20% glycerol, 40 mM uridine and was flash-cooled in liquid nitrogen.

Crystallisation of Pac13 mutants and soaking with uridine.

Pac13-His_8 Y55F, Pac13-His_8 Y89F and Pac13-His_8 H42Q at 6.6, 4.5 and 5.5mg/ml respectively, in 25 mM Tris-base [tris (hydroxymethyl) aminomethane] pH 7.9, 100 mM NaCl were used for crystallisation. Microseeding with Pac13-His_8 wt was employed so as to ensure crystallisation of the mutants. Crystallisation was achieved using drops containing 100 nL protein, 90 nL well solution (appropriate for each mutant) and 10 nL seed solution in sitting drop MRC plates at 20 °C. Well solution utilised: 0.1 M MES [2-(*N*-morpholino)ethanesulfonic acid] pH 6.5, 0.2 M ammonium sulfate, 30 % w/v poly[ethylene glycol] [PEG]- 5 K MME (for Pac13-His_8 Y55F), 0.2 M ammonium fluoride, 20 % w/v poly[ethylene glycol] [PEG]-3350 (for Pac13-His_8 Y89F) and 0.2 M potassium sodium tartarate, 20 % w/v poly[ethylene glycol] [PEG]-3350 (for Pac13-His_8 H42Q). The seed stock solution was prepared from a Pac13-His8 wt crystal (growth conditions: 0.1 M BisTris propane [2,2-Bis(hydroxymethyl)-2,2',2''-nitrilotriethanol] pH 7.5, 0.2 M Sodium acetate, 20% w/v poly[ethylene glycol] [PEG]-3350) using the Seed Beads, according to manufacturer's procedures. Soaking was performed in 24-well MRC plates (300 µl well volume), using the hanging-drop method. A single crystal was used for soaking in a 2 µl drop, containing mother liquor supplemented with uridine **7** (stock in MQ water), in a final concentration of 0.08 M (for Pac13-His_8 H42Q) and 0.1 M (for Pac13-His_8 Y55F). A single crystal of Pac13-His_8 Y89F was used for soaking in a 3 µl drop, containing mother liquor supplemented with uridine **7** (stock in MQ water), in a final concentration of 0.133 M. Following incubation for 3 h at a 20 ºC incubator, the crystals were cryo-protected in mother liquor containing 20% ethylene glycol, and appropriate uridine concentration (40 mM for Pac13-His_8 Y89F, Pac13-His_8 H42Q and 50 mM for Pac13-His_8 Y55F) and were flash-cooled in liquid nitrogen.

Data collection, processing and refinement

Data collection of Pac13 wt and Pac13 SeMet.

X-ray diffraction datasets of w.t and SeMet Pac13 were collected at 100 K at the Diamond Light Source Beamline I03 with SeMet Pac13 collected at the Se-K absorption edge. The data were processed and scaled using xia2 (Winter, 2010), utilising XDS (Kabsch, 2010), AIMLESS (Evans & Murshudov, 2013) and the CCP4 suite of programs (Winn *et al*., 2011). Additionally, SeMet Pac13 was processed using PHENIX AutoSol and AutoBuild (Adams *et al*, 2010). The crystal space group is $P3_221$ with a single molecule in the asymmetric unit. The final structures were refined using REFMAC5 *(G.N.Murshudov* et al. *1999).* Data collection and refinement statistics are given in Table S2. The coordinates and structure factors have been deposited in the Protein Data Bank under the accession code 5NJN.

Table S2. Data collection, processing and refinement.

	Native Pac13	SeMet Pac13
Data collection and Processing		
Diffraction source	Diamond Light Source Beamline I03	Diamond Light Source Beamline I03

Wavelength (Å)	0.97970	0.97945
Temperature (K)	100.0	100.0
Detector	PILATUS 6M detector	PILATUS 6M detector
Space group	P3221	P3221
a, b, c (Å)	66.5, 66.5, 54.7	66.8, 66.8, 54.7
α, β, γ (°)	90.0, 90.0, 120.0	90.0, 90.0, 120.0
Resolution range (Å)	57.58 – 1.55 (1.61 – 1.55)	57.85 – 1.60 (1.64 – 1.60)
Total No. of reflections	224546 (21509)	598308 (41220)
No. of unique reflections	20630 (2031)	19005 (1384)
Completeness (%)	100.0 (99.9)	100.0 (100.0)
Redundancy	10.9 (10.6)	31.5 (29.8)
Anomalous Completeness (%)	100.0 (99.6)	100.0 (100.0)
Anomalous Redundancy	5.6 (5.2)	16.3 (15.1)
⟨ I/σ(I)⟩	23.5 (3.6)	32.7 (6.2)
Rmeas.	0.061 (0.817)	0.096 (0.772)
Overall B factor from Wilson plot (Å2)	18.63	14.75
Refinement		
No. of reflections, working set	20654	18984
No. of reflections, test set	1056	979
Final R_{cryst}	0.1799	0.1845
Final R_{free}	0.2011	0.2066
No. of non-H atoms	1157	1073
Protein	976	968
Water	181	105
R.m.s. deviations		
Bonds (Å)	0.006	0.006
Angles (°)	1.27	1.30
Average *B* factors (Å^2)	23.6	18.7
Protein	20.5	17.6
Water	30.9	29.1

X-ray Methods for Complexes

X-ray diffraction data were collected at 100 K on a FRE+/A200 home source. The data were processed and scaled using autoPROC (Vonrhein *et al.*, 2011), utilising XDS (Kabsch, 2010), AIMLESS (Evans & Murshudov, 2013) and the CCP4 suite of programs (Winn *et al.*, 2011). The crystal space group is P$3_2$21 with a single molecule in the asymmetric unit. Data collection statistics are given in Table S3.

The structures were determined using the coordinates of an isomorphous unliganded protein model, with preliminary refinement carried out using autoBUSTER (Bricogne *et al.*, 2014) or REFMAC5 (G.N.Murshudov *et al.* 1999). In all cases, the ligands were clearly visible in the resulting F_o-F_c electron density maps (Table S4). Coot (Emsley *et al.*, 2010) was used for model building, with refinement completed using autoBUSTER. The statistics for the final models are given in Table S3. The coordinates and structure factors have been deposited in the Protein Data Bank under the accession codes 5OO4, 5OO5, 5OO8, 5OO9 and 5OOA..

Table S3. Data collection, processing and refinement of Pac13 complexes

	Native Pac13 - uridine	Native Pac13 – uridine 5′-uronic acid	Mutant Pac13 H42Q - uridine	Mutant Pac13 Y55F - uridine	Mutant Pac13 Y89F - uridine
Protein	25mM Tris, pH 7.9, 100mM NaCl @1.8mg/mL	25mM Tris, pH 7.9, 100mM NaCl @1.6mg/mL	25mM Tris, pH 7.9, 100mM NaCl @5.5mg/mL	25mM Tris, pH 7.9, 100mM NaCl @6.6mg/mL	25mM Tris, pH 7.9, 100mM NaCl @4.5mg/mL
Crystallisation conditions	15% PEG3350,0.2M NaAc,0.09MBis-Tris pH6.5	16% PEG3350,0.1M $MgCl_2$,0.2MBis-Tris pH8.8	0.2 M potassium sodium tartrate, 20 % w/v PEG-3350	0.1 M MES pH 6.5, 0.2 M NH_4SO_4, 30 % w/v 5K PEGMME	0.2 M ammonium fluoride, 20 % w/v PEG-3350
Diffraction source	In house FRE+/A200	In house FRE+/A200	In house FRE+/A200	In house FRE+/A200	In house FRE+/A200
Wavelength (Å)	1.54178	1.54178	1.54178	1.54178	1.54178
Temperature (K)	100.0	100.0	100.0	100.0	100.0
Detector	Rigaku Saturn A200	Rigaku Saturn A200	Rigaku Saturn A200	Rigaku Saturn A200	Rigaku Saturn A200
Space group	$P3_221$	$P3_221$	$P3_221$	$P3_221$	$P3_221$
a, *b*, *c* (Å)	66.8, 66.8, 55.0	66.5, 66.5, 55.2	66.4, 66.4, 55.8	66.6, 66.6, 54.3	66.8, 66.8, 54.6
α, β, γ (°)	90.0, 90.0, 120.0	90.0, 90.0, 120.0	90.0, 90.0, 120.0	90.0, 90.0, 120.0	90.0, 90.0, 120.0
Resolution range (Å)	28.93 – 1.60 (1.71 – 1.60)	57.85 – 1.78 (1.93 – 1.73)	39.68 – 1.78 (1.92 – 1.78)	39.52 – 1.59 (1.68 – 1.59)	39.69 – 1.60 (1.71 – 1.60)
Total No. of reflections	62546 (4304)	50786 (9834)	49057 (9547)	67027 (3557)	59333 (4310)
No. of unique reflections	17785 (2281)	13723 (2788)	13630 (2778)	17643 (2002)	17880 (2560)
Mn(I) CC(1/2)		0.998 (0.825)	0.998 (0.745)	0.999 (0.820)	0.998 (0.858)
Completeness (%)	92.5 (67.1)	99.3 (99.3)	99.5 (99.7)	92.8 (67.5)	95.3 (76.8)
Redundancy	3.5 (1.9)	3.7 (3.5)	3.6 (3.4)	3.8 (1.8)	3.3 (1.7)
$\langle I/\sigma(I)\rangle$	27.4 (7.0)	19.4 (5.0)	19.2 (3.4)	42.2 (8.4)	37.3 (7.6)
$R_{meas.}$	0.027 (0.108)	0.050 (0.248)	0.051 (0.376)	0.018 (0.096)	0.019 (0.107)
Overall *B* factor from Wilson plot ($Å^2$)	13.848	16.66	21.25	15.20	15.15
Refinement					
No. of reflections, working set	16828	12994	12900	16782	16947
No. of reflections, test set	838	673	672	837	847
Final R_{cryst}	0.1572	0.1531	0.1624	0.1601	0.158
Final R_{free}	0.1846	0.1951	0.1916	0.2000	0.186
No. of non-H atoms	1297	1287	1180	1248	1244
Protein	1057	1061	1018	1041	1030
Water	223	208	141	190	197
R.m.s. deviations					
Bonds (Å)	0.006	0.006	0.005	0.005	0.007
Angles (°)	1.31	1.22	1.23	1.22	1.26
Average *B* factors ($Å^2$)	15.3	17.9	22.5	16.5	16.5
Protein	12.3	15.4	20.5	14.1	14.0
Water	29.5	30.9	37.6	29.7	30.2
Ligand/Other	14.8	13.6	19.0/29.4	12.9	12.1

Table S4. A Difference density map (fofc) for ligands contoured at ±3sigma (green, red); **B** fofc electron density map overlaid with 2fofc map contoured at +1sigma (blue) centred around mutated residue.

	Native Pac13 - uridine	Native Pac13 – uridine 5′-uronic acid	Mutant Pac13 H42Q - uridine	Mutant Pac13 Y55F - uridine	Mutant Pac13 Y89F - uridine
**A**	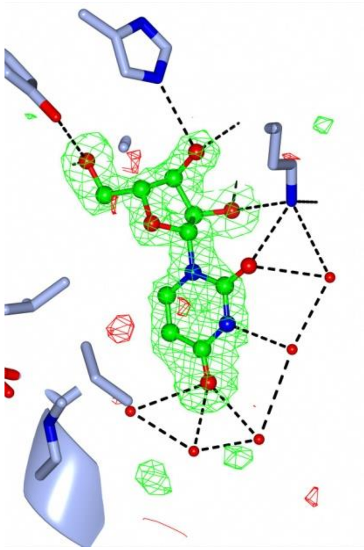	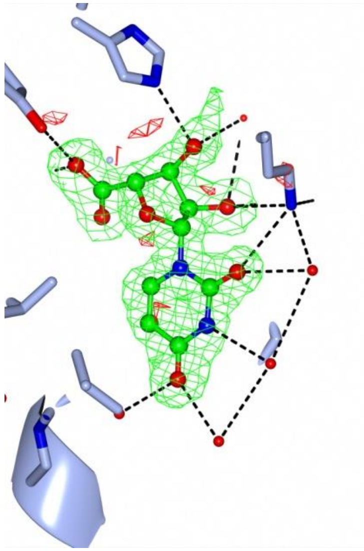	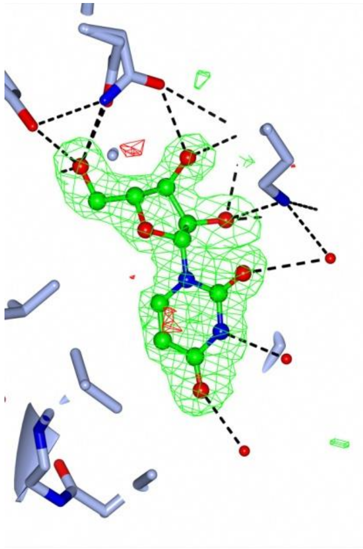	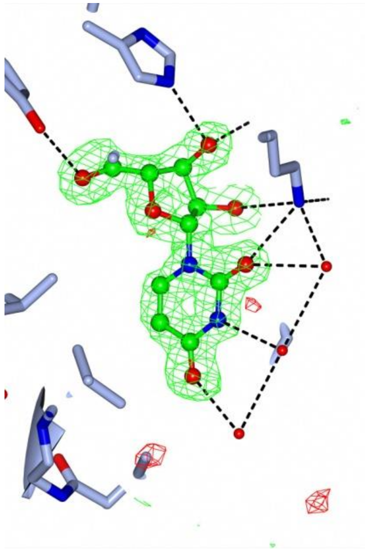	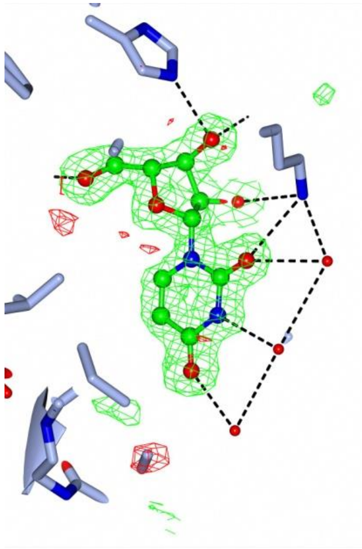
**B**			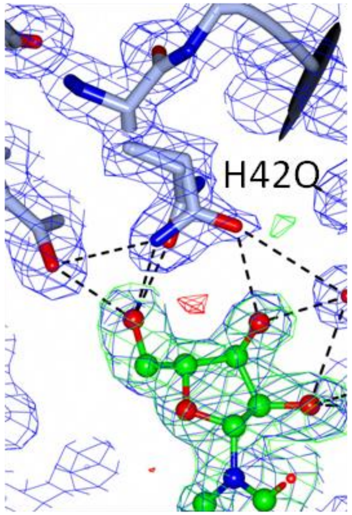	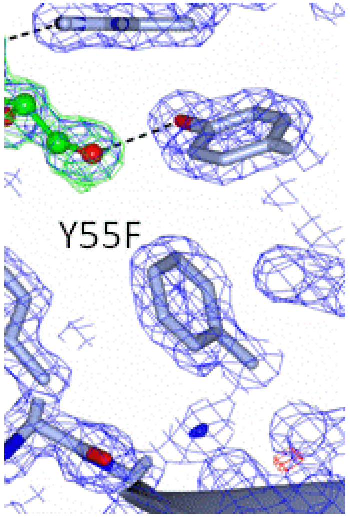	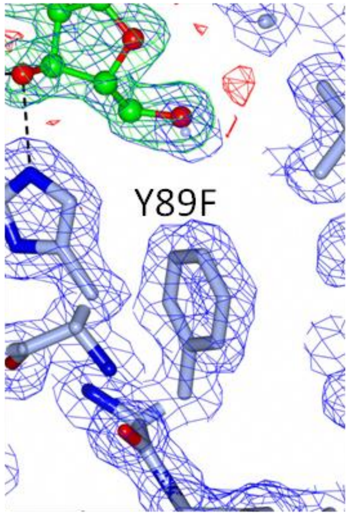

Synthesis of uridine-5′-aldehyde, 1.
The synthesis of **1** was a modification of previously described procedures.[1]

*2′,3′-O-Isopropylidene uridine **14**.* Uridine **7** (500 mg, 2 mmol) was dissolved in acetonitrile (30 ml). 2, 2-dimethoxypropane (2 ml, 16 mmol) and *p*-toluenesulfonic acid (15 mg) were added and the mixture was refluxed for 3 h. The reaction mixture was concentrated under vacuum to give a brown residue. The residue was purified by flash chromatography (100% DCM to 100% acetone in a stepwise gradient) to give the desired compound **14** in a 94% yield. ^{1}H-NMR (MeOD, 500 MHz) δ 7.86 (d, J = 8.1 Hz, 1H), 5.89 (d, J = 3.0 Hz, 1H), 5.71 (d, J =8.1 Hz, 1H), 4.93 (dd, J = 6.6, 3.2 Hz, 1H), 4.84 (dd, J = 6.5, 3.4 Hz, 1H), 4.23 (m, 1H), 3.79 (dd, J = 12, 3.6 Hz, 1H), 3.73 (dd, J = 12.2, 3.8 Hz, 1H), 1.57 (s, 3H), 1.38 (s, 3H). ^{13}C-NMR-DEPTQ (MeOD, 126 MHz) δ (ppm) 164.8, 150.6, 142.4, 113.7, 101.2, 92.7, 86.9, 84.4, 80.8, 61.6, 26.1, 24.1. MS(ES^{+}) m/z 285.1 ([M + H]$^{+}$, 100%), 569.2 ([2M + H]$^{+}$, 67%), HRMS (ES^{+}) m/z calc. for $C_{12}H_{17}N_{2}O_{6}$ [M + H]$^{+}$ 285.1081, found 285.1075.

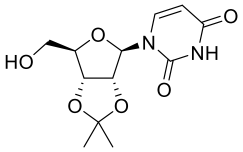

14

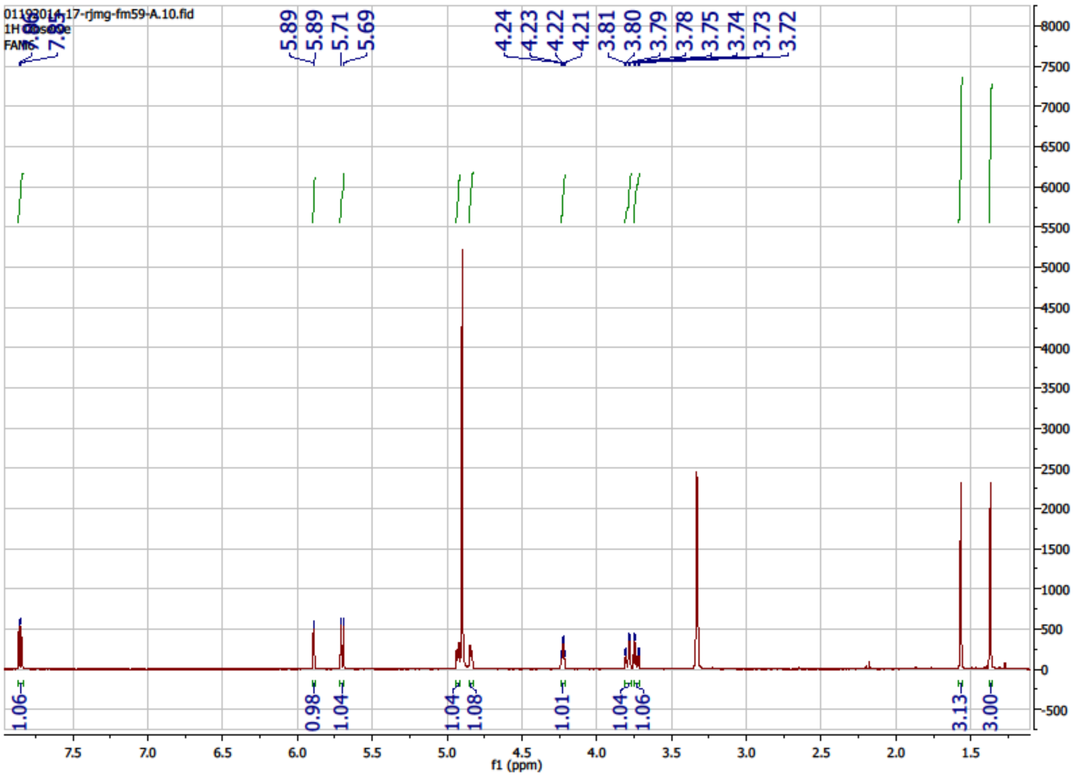

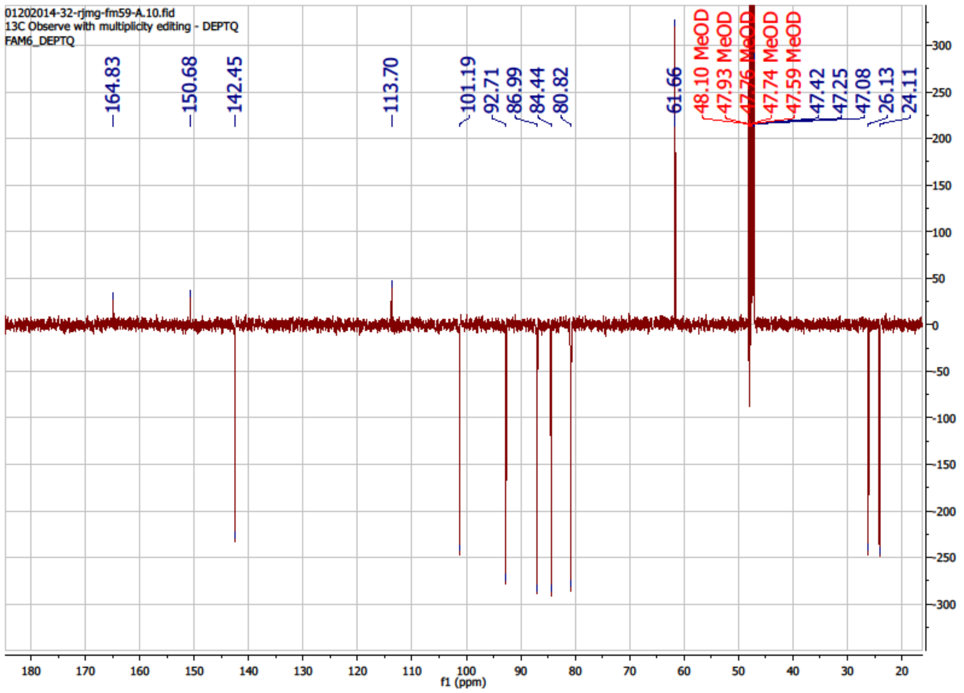

Figure S6. ^{1}H and ^{13}C (DEPTQ) NMR of compound **14**

2′, 3′-O-Isopropylidene-5′-deoxy-5′-uridylaldehyde ***15*** 2′, 3′-*O*-isopropylidene uridine **14** (284 mg, 1 mmol) was dissolved in acetonitrile (30 ml). IBX (2-iodoxybenzoic acid) (830 mg, 3 mmol) was then added and the solution was refluxed. The reaction was monitored by TLC (10% ethanol in chloroform) and when judged complete, after the course of 5 h, the reaction was stopped. The mixture was allowed to cool down to room temperature. The precipitated IBX was removed by filtration. The filtrate was washed with water and the organic phase was concentrated under vacuum. The residue was purified by flash chromatography (100% DCM to 100% acetone in a stepwise gradient) to yield the title compound **15** as an off-white solid (197 mg, 70% yield). ^{1}H NMR ($CDCl_3$, 300 MHz) δ 9.43 (s, 1H), 8.50 (s, 1H), 5.76 (dd, J = 8.0, 2.2 Hz, 1H), 5.48 (s, 1H), 5.21 (dd, J = 6.3, 1.6 Hz, 1H), 5.09 (d, J = 6.3 Hz, 1H), 4.56 (d, J = 1.6 Hz, 1H), 1.53 (s, 3H), 1.36 (s, 3H). ^{13}C-NMR-DEPTQ ($CDCl_3$, 126 MHz) δ 199.3, 162.7, 150.4, 144.1, 103.0, 100.4, 94.2, 85.0, 83.8, 26.6, 24.9. MS(ES$^+$) *m/z* 283.1 ($[M + H]^+$, 100%), HRMS (ES$^+$) calc. for $C_{12}H_{17}N_2O_6$ $[M + H]^+$ 283.0925, found 283.0927.

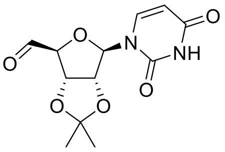

15

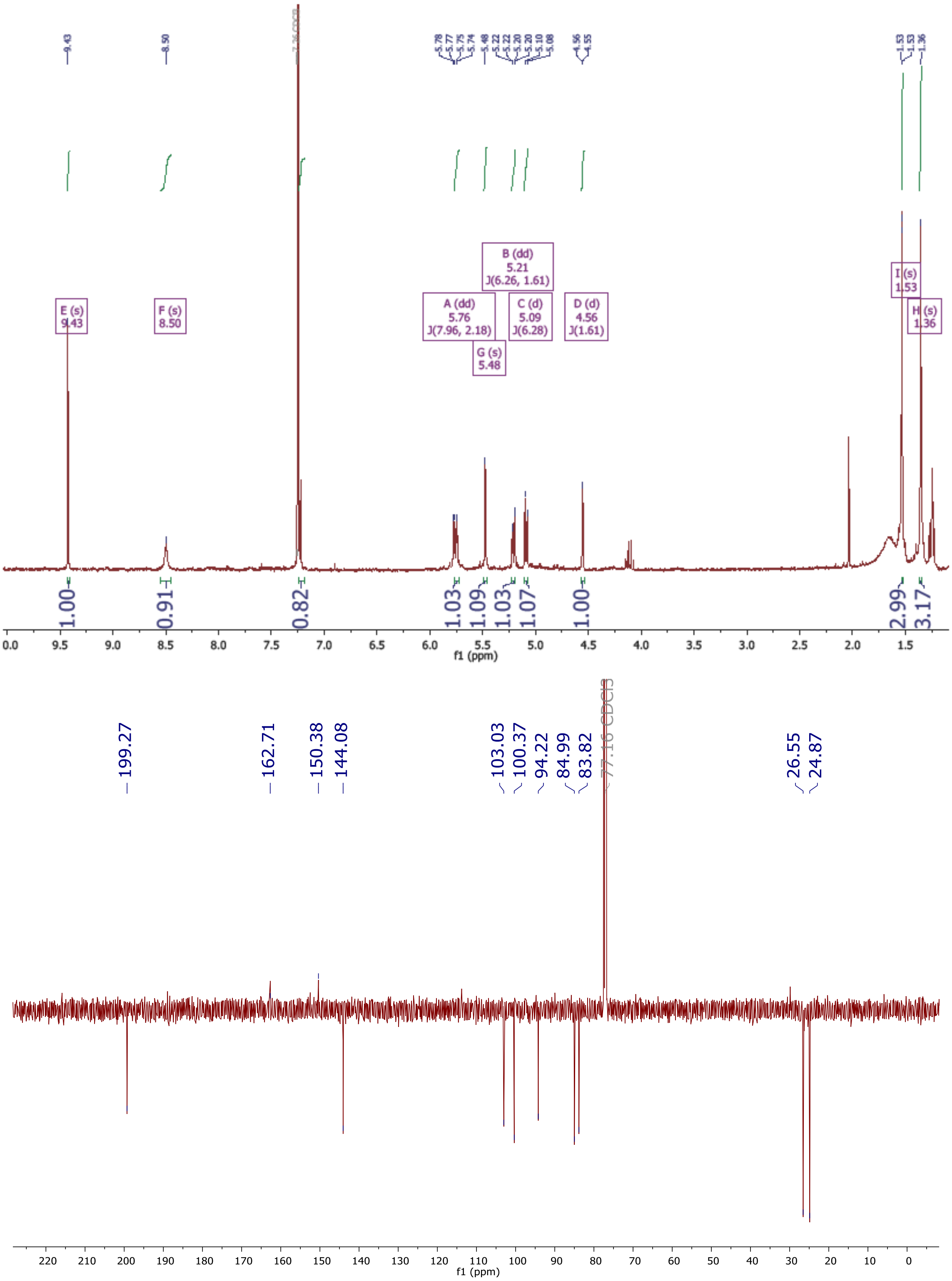

Figure S7. ^{1}H and ^{13}C (DEPTQ) NMR of compound **15.**

*Uridine-5'-aldehyde **1**.* A solution of **15** (40 mg, 0.17 mmol) in 9:1 trifluoroacetic acid-water (0.4 ml) was stirred at 4 ºC (ice bath) for 45 min until completion (monitored by TLC). The reaction mixture was then diluted with water (5 ml) and the resulting solution was extracted with ethylacetate (2 X 5 ml). The aqueous phase was freeze-dried overnight. The resulting material was obtained as a hydroscopic powder in a 90% yield (33 mg) and stored at -80 ºC. In aqueous conditions, the compound exists solely as the hydrate[1a]. When judged necessary, the material was further purified by HPLC method B. The data were in accordance with previous data in the literature[1a, 1b]. ^{1}H NMR (D_2O, 400 MHz) δ 7.93 (d, J = 8.2 Hz, 1H), 6.01 (d, J = 6.2 Hz, 1H), 5.94 (d, J = 8.1 Hz, 1H), 5.23

(d, J = 3.9 Hz, 1H), 4.43 (t, J = 5.8 Hz, 1H), 4.35 (t, J = 4.4 Hz, 1H), 4.06 (apt, J = 3.7 Hz, 1H). ^{13}C-NMR-DEPTQ (D_2O, 126 MHz) δ 166.1, 151.8, 141.9, 102.5, 88.6, 88.4, 86.2, 73.3, 69.7. MS(ES$^+$) m/z 243.0 ([M + H]$^+$, 100 %).

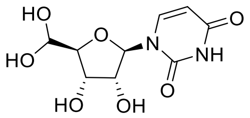

1

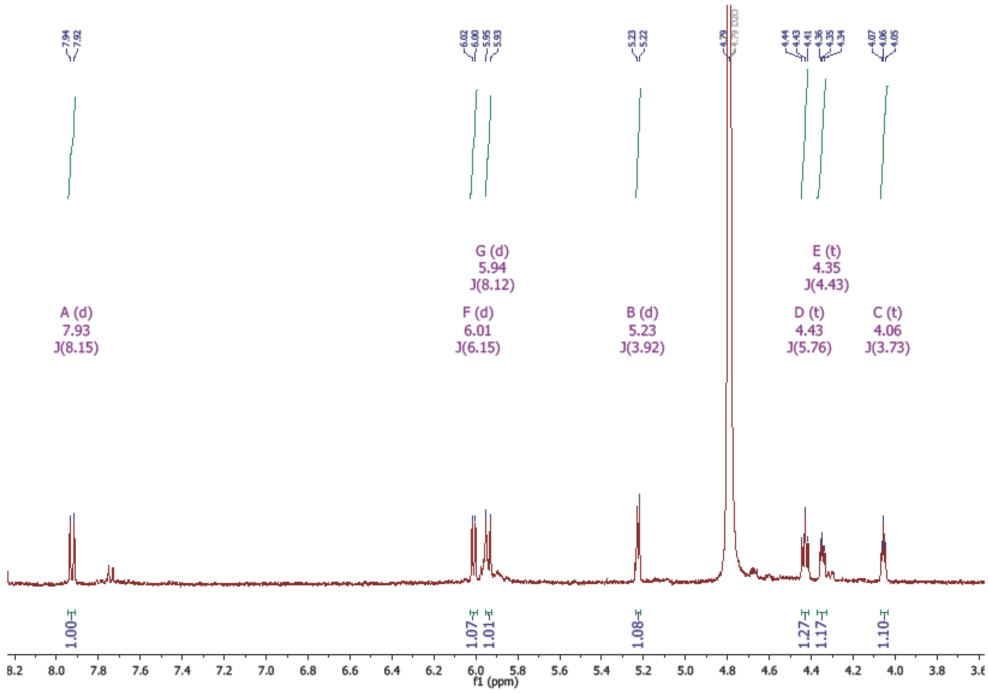

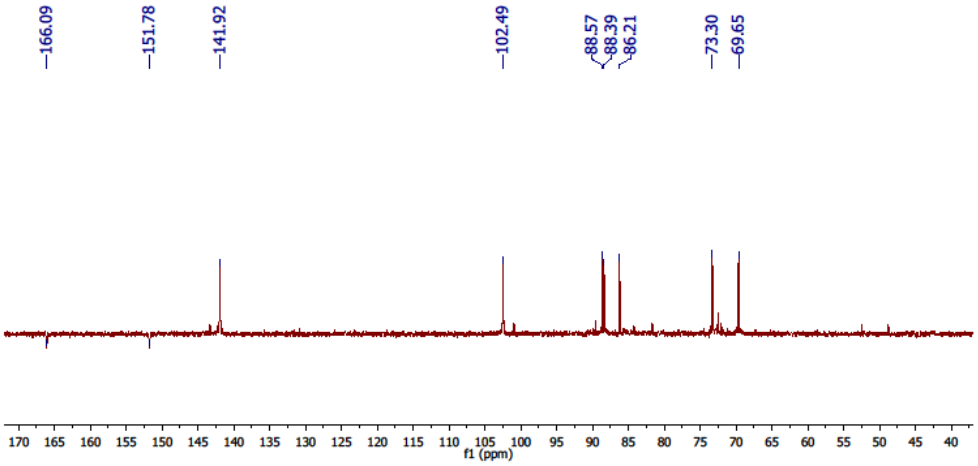

Figure S8. ^{1}H and ^{13}C (DEPTQ) NMR of compound **1.**

Uridine-5′-uronic acid 8.

Compound **8** was obtained after gradual oxidation of compound **1**, after exposure of compound **1** (0.1 M) in a solution of. 0.1 M HEPES [4-(2-hydroxyethyl)-1-piperazineethanesulfonic acid] pH 7.4 after a period of 15 days and aliquotes were analysed by LC-MS.The LC-MS data were identical with the data obtained for a GSK synthetic standard of uridine-5′-uronic acid, for which ^{1}H NMR data were also available.

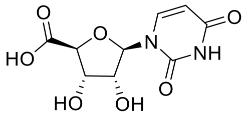

8

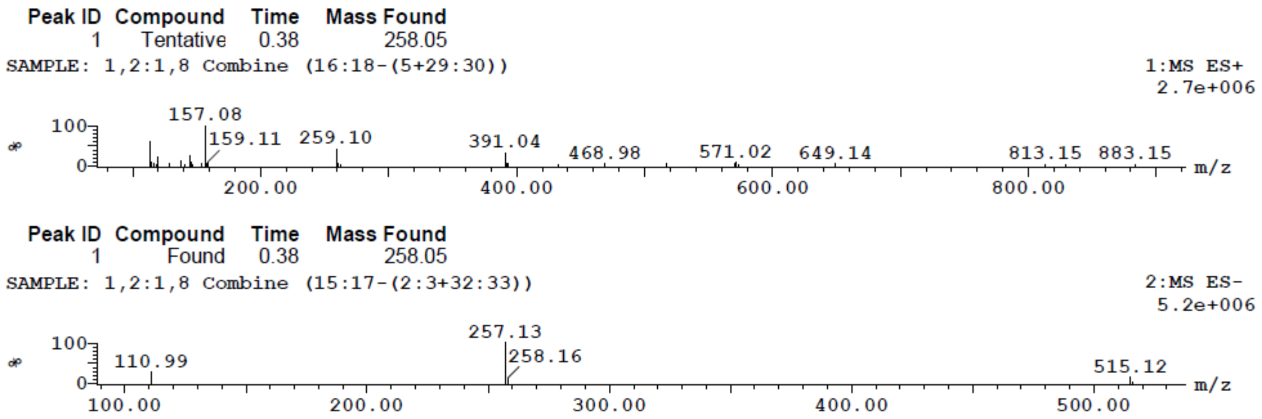

Figure S9. LC-MS of synthetic standard of uridine-5-uronic acid **8**.

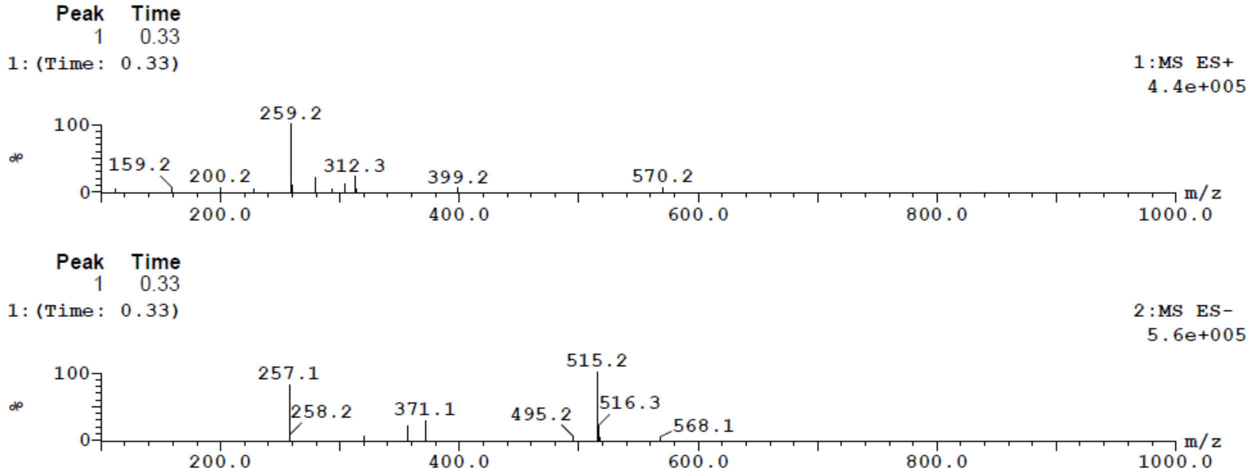

Figure S10. LC-MS of uridine-5-uronic acid **8**, used for soaking into Pac13 crystalls.

NMR assay of Pac13 with uridine-5′-aldehyde 1

NMR assay at pD 9

A buffer containing 100 mM NaH_2PO_4 was prepared in D_2O and the pD was adjusted to 9 with NaOH. Pac13 wt (1.5 mg/ml) 500 µl was diluted 3X in the 100 mM NaH_2PO_4, pD 7.5 buffer in D_2O and was spin-concentrated to 50 µl using an Amicon Ultra-0.5 ml centrifugal filter (3 K MWCO) according to manufacturer’s procedures. Freshly prepared uridine-5′-aldehyde **1** (2 mg) was dissolved in 550 µl of buffer and were combined with the 50 µl of Pac13. Another sample containing only uridine-5′-aldehyde **1** (2 mg) was prepared as the negative control. ^{1}H NMR (600 MHz) spectrum using solvent suppression was recorded at several time points for the two samples.

NMR assay at pD 7.5

A buffer containing 100 mM NaH_2PO_4 was prepared in D_2O and the pD was adjusted to 7.5 with NaOH. Pac13 wt (1.5 mg/ml) 590 µl

was diluted 3X in the 100 mM NaH_2PO_4, pD 7.5 buffer in D_2O and was spinned-concentrated to 200 µl using an Amicon Ultra-0.5 ml centrifugal filter (3 K MWCO) according to manufacturer's procedures. Freshly purified uridine-5′-aldehyde **1** (2.1 mg) was dissolved in 550 µl of buffer and were combined with the 200 µl of Pac13. An ^{1}H NMR (500 MHz) spectrum using solvent suppression was recorded at several time points. The structure of Pac13 product was assigned by COSY and HSQC (500 MHz).

3′-Deoxy-3′,4′-didehydrouridine-5′-aldehyde. ^{1}H NMR (D_2O, 500 MHz) *δ* 7.38 (d, *J* = 8.0 Hz, 1H), 6.24 (d, *J* = 2.2 Hz, 1H), 5.80 (d, *J* = 8.1 Hz, 1H), 5.58 (s, 1H), 5.44 (dd, *J* = 2.7, 0.9 Hz, 1H), 5.00 (m, 1H). HSQC (D_2O, 126 MHz) *δ* 140.1, 102.0, 99.1, 93.0, 84.0, 77.0. MS(ES$^+$) *m/z* 113.0 ([M –$C_5H_4O_3$]$^+$, 100 %), 225.0 ([M + H]$^+$, 65 %).

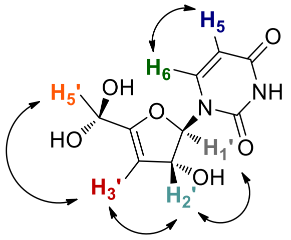

2b

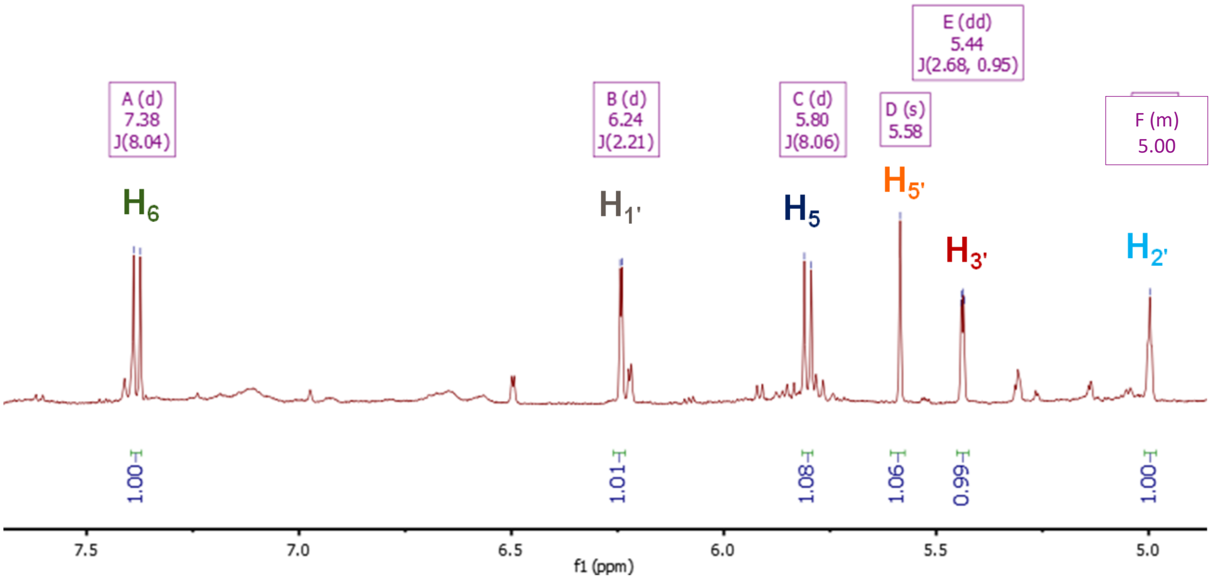

Figure S11. ^{1}H NMR of compound **2.**

An NMR spectrum for **2b** in D_2O has not been reported in the literature. However, the NMR spectra of compound **2,** as well as of structurally related compound, recorded in d_6-DMSO (under which conditions the compound exists in the aldehyde form) have been reported in the literature.[2]

HPLC assays, steady-state kinetic and pH profile analysis

All assays were performed in triplicates. Alongside the assays, negative controls excluding the active enzyme or component, were carried out for each assay in triplicates. The reactions were stopped by addition of equal volume of 1% TFA and the protein was removed by centrifugation (4000 rpm, 10 °C, 10 min) or filtration over Acroprep 96 filter plates 0.45 µm (Merck Millipore) according to manufacturer's procedures. The samples were typically analysed by HPLC method A and/or LC-MS method C.

Assay in presence of EDTA

Pac13 wt (20 µM) and EDTA (50 and 100 mM) were incubated with gentle agitation in 100 mM HEPES pH 7.5 buffer, in a final volume of 50 µl, for 1 h at ambient temperature. Following the 1h incubation, substrate uridine-5′-aldehyde **1** (1 mM) was added and the reaction was incubated at 30 °C for 15 h. A positive control, excluding the presence of EDTA and appropriate negative controls, excluding the presence of enzyme were carried out under the same conditions.

Assay in presence of NADPH, NADH, $MgCl_2$

Pac13 wt and Pac13-His_8 wt (10 µM) and substrate uridine-5′-aldehyde **1** (1 mM) were incubated separately in 100 mM HEPES pH 7.5 and 100 mM NaH_2PO_4 pH 7.9, in a final volume of 50 µl at 40 °C for 5 h. NADH, NADPH and $MgCl_2$ were added to each assay at a final concentration of 200 µM, 200 µM and 20 mM, respectively. Positive controls, excluding the presence of NADPH, NADH, $MgCl_2$ respectively, and appropriate negative controls, excluding the presence of enzyme were carried out under the same conditions.

pH profile of Pac13

Pac13-His_8 wt (16 µM) and substrate uridine-5′-aldehyde **1** (1 mM) were incubated in 100 mM citrates-phosphates-borates buffer of a varied pH (5.0 – 10.5, with a 0.5 interval) in a final volume of 50 µl at 40 °C for 6 h. The % conversion calculated from peak integration was plotted against pH values to construct a bell-shaped curve. The pka values were calculated using GraphPad.

Kinetic analysis of Pac13 wt and mutants

Pac13-His_8 wt (5 µM), Pac13-His_8 Y55F (15 µM), Pac13-His_8 K16R (15 µM) and substrate uridine-5′-aldehyde **1** in appropriate concentrations (0.0, 0.1, 0.25, 0.5, 1.0, 1.25, 2.5, 5.0, 7.5, 10 mM for reactions with wt and 0.0, 0.078, 0.156, 0.312, 0.625, 1.25, 2.5, 5, 7.5, 10 mM for reactions with K16R or Y55F) were incubated in 100 mM Na_2HPO_4 pH 7.8 at 40 °C for 135 min. The pH of the substrate stock was adjusted to 7.8 beforehand. Negative controls, excluding the presence of the enzymes, were carried out for the three time points. The reactions were immediately stopped by addition of 1% TFA and filtration and were analysed with HPLC method A. Initial rates of reaction were calculated from peak integration and used to construct a Michaelis-Menten curve using Origin.

Assays with Pac13 mutants

Pac13-His_8 wt, Pac13-His_8 K16R, Pac13-His_8 H42Q, Pac13-His_8 E108Q, Pac13-His_8 Y55F and Pac13-His_8 Y89F (10 µM) and substrate uridine-5′-aldehyde **1** (1 mM) were incubated in 100 mM Na_2HPO_4 pH 7.8 at 40 °C in a final volume of 50 µl, for 0, 3 and 20 h. Negative controls, excluding the presence of the enzymes, were carried out for the three timepoints. The reactions were stopped as usual and analysed with HPLC method A.

HPLC method A

The following method was used for monitoring the Pac13 enzymatic reaction. Column: Atlantis dC18, 1 mm*150 mm, 3 µm (Waters, UK). Buffer A: 100 % 20 mM aqueous ammonium formate pH 3.0, buffer B: 100 % CH_3CN. Injection volume: 5 µl. Temperature: 25 ºC. UV detection: 260 nm and 280 nm. Gradient profile:

Time (minutes)	Flow rate (ml/min)	% B
0.0	0.1	0
6.0	0.1	0
7.0	0.1	50
9.0	0.1	50
10.0	0.1	0
16.0	0.1	0

Substrate	Product
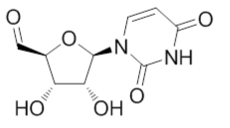	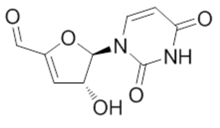
Rt: 2.3 min	Rt: 4.1 min

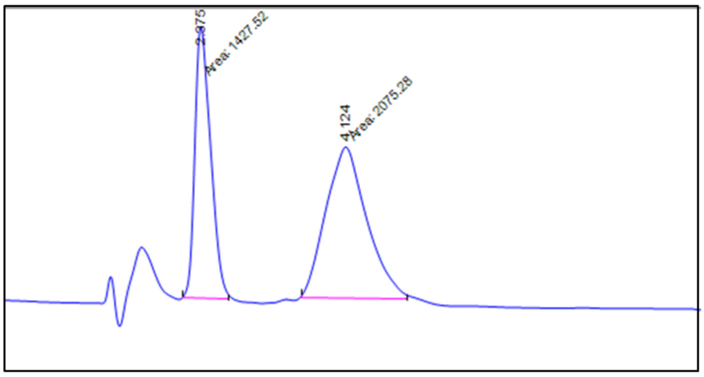

Figure S12. HPLC trace of Pac13 assay using Method A.

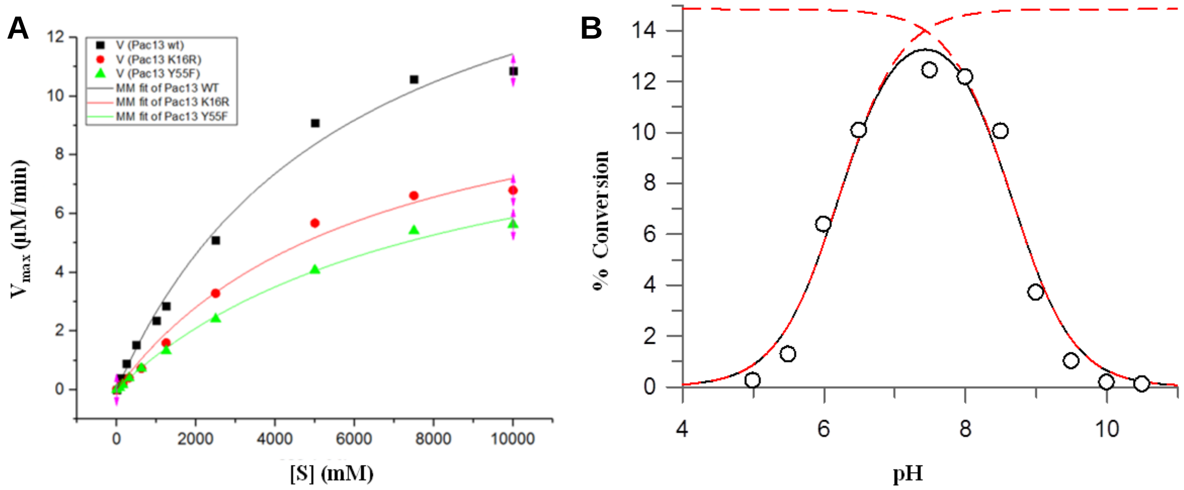

Figure S13. A Kinetic analysis of Pac13 WT, K16R and Y55F. **B** pH profile for Pac13.

LC-MS analysis of enzymatic assays

LC-MS method C

The following method was used for monitoring the Pac13 assay. Column: Acquity UPLC, T3 HSS, 50 mm*2.1 mm, 1.8 µm (Waters, UK). Buffer A: 0.1 % formic acid in MQ H_2O, buffer B: 100 % CH_3OH. Injection volume: 5 µl. Temperature: 40 ºC. UV detection: 254 nm. Gradient profile:

Time (minutes)	Flow rate (ml/min)	% B
0.0	0.35	0
1.5	0.35	0
6.5	0.35	60
8.5	0.35	60
9.0	0.35	0
11.0	0.35	0

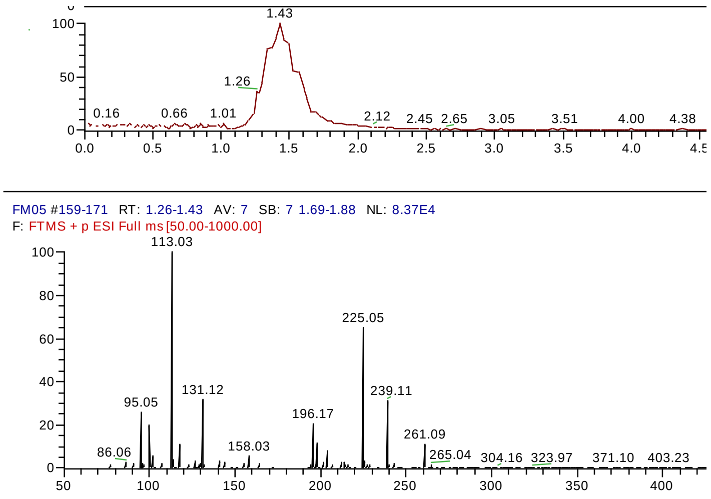

Figure S14. LC-MS analysis of Pac13 assay with aldehyde; extracted ion for *m/z* 225, corresponding to $[M+H]^+$ of compound **2**.

HPLC purification of uridine-5′-aldehyde

HPLC method B

The following method was used for the purification of uridine-5′-aldehyde. Column: Atlantis Hilic Prep 19 mm*150 mm, 5 µm (Waters, UK). Buffer A: 30 % 16.7 mM aqueous ammonium formate pH 3.0 in CH_3CN, buffer B: 5 % 100 mM aqueous ammonium formate pH 3.0 in CH_3CN. Injection volume: 500 µl. Temperature: ambient. UV detection: 257 – 400 nm (averaged). The fractions that corresponded to the pure product were combined and lyophilised overnight. Gradient profile:

Time (minutes)	Flow rate (ml/min)	% B

0.0	20.00	100
0.5	20.00	100
16.0	20.00	76
22.5	20.00	76
23	20.00	100
30	20.00	100

Results and Discussion

Dehydratases: an overview of the main classes

In nature, dehydratases are a diverse array of enzymes that span a plethora of structural and mechanistic possibilities. Up till now, characterized dehydratases include those that are co-factor and metal-dependent as well as independent enzymes [3] employing metal-dependent, acid-base, radical [4] and covalent mechanisms [3] (Figure S15). RmlB [5], perhaps one of the most studied and ubiquitous carbohydrate-active lyases, is a dTDP-$_D$-glucose 4, 6-dehydratase that shares significant similarities to guanosine diphosphate mannose (GDP)-$_D$-mannose 4, 6- dehydratase [6] (GMD) and the UDP-Glc*N*Ac 5, 6-dehydratase TunA [7]. These enzymes are members of the short chain dehydrogenase/reductase superfamily (SDR) and operate through a similar mechanism, initiated by the NAD^+ assisted oxidation of a hydroxyl group within the substrate, followed by elimination of water to generate an enone then reduction of the resultant conjugated *C=C* or *C=O* double bond (Figure S16). RmlB from *Salmonella enterica* and *Streptococus suis* have been crystallized as homodimers of 40.7 kDa and 38.9 kDa per subunit respectively [5c], while GMD enzymes, including the GMD from *Pseudomonas aeruginosa,* have been mostly reported as homotetramers [6].

Another common dehydratase present in a variety of organisms is 3-dehydroquinate dehydratase (DHQ) [8] which catalyses the third step of the shikimate pathway. DHQ-type enzymes can be subdivided into two classes, I and II which exhibit two distinct mechanisms. DHQ II catalyzes the reversible anti elimination of water through an E1cB mechanism that proceeds via enolate formation (Figure S16). In contrast to Pac13, DHQ II from *M. tuberculosis* and *H. pylori* abstract the acidic proton via a conserved tyrosine, while a conserved histidine is the active site acid, assisting the acid-catalysed removal of the leaving hydroxyl. In contrast, DHQI goes through a multistep mechanism, including Schiff base formation with a conserved lysine (Figure S16). DHQI enzymes have been reported as dimers of 27 kDa per subunit, whereas DHQII from *Streptomyces coelicolor* and *Mycobacteria tuberculosis* are dodecameric [3, 5b, 8a, 8b, 9]. The linalool dehydratase-isomerase (LinD) [10] is a co-factor independent enzyme that has been proposed to employ acid-base catalysis for the protonation of the leaving hydroxyl group of linalool and dehydration at the chiral carbon, mediated by residues C171, Y45 and N39. Although previously reported as tetrameric in solution, LinD has been crystallized as a pentamer, comprising an α/α_6 barrel per monomer [10].

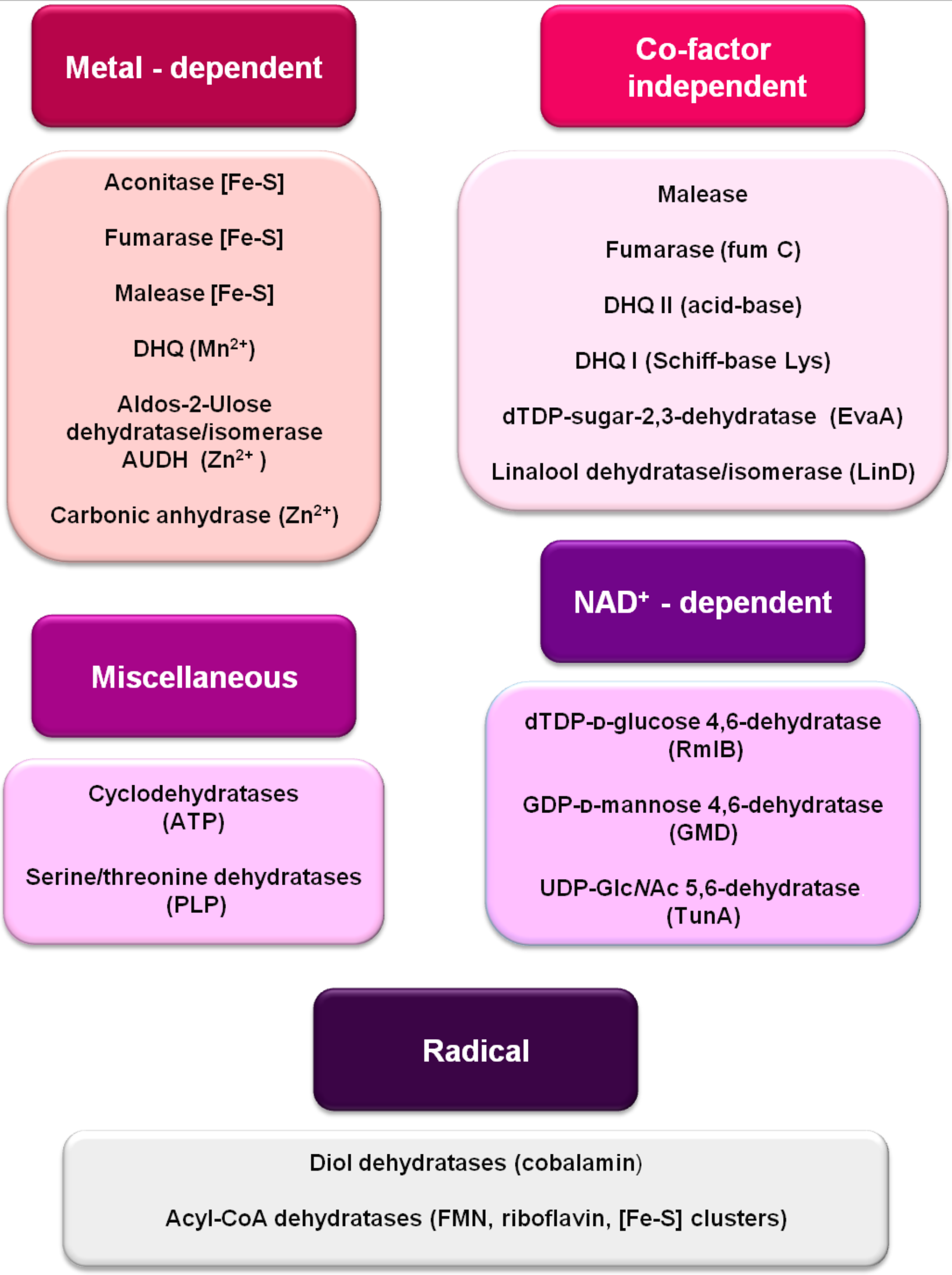

Figure S15. Classification of dehydratases based on their mechanism/co-factor dependency.

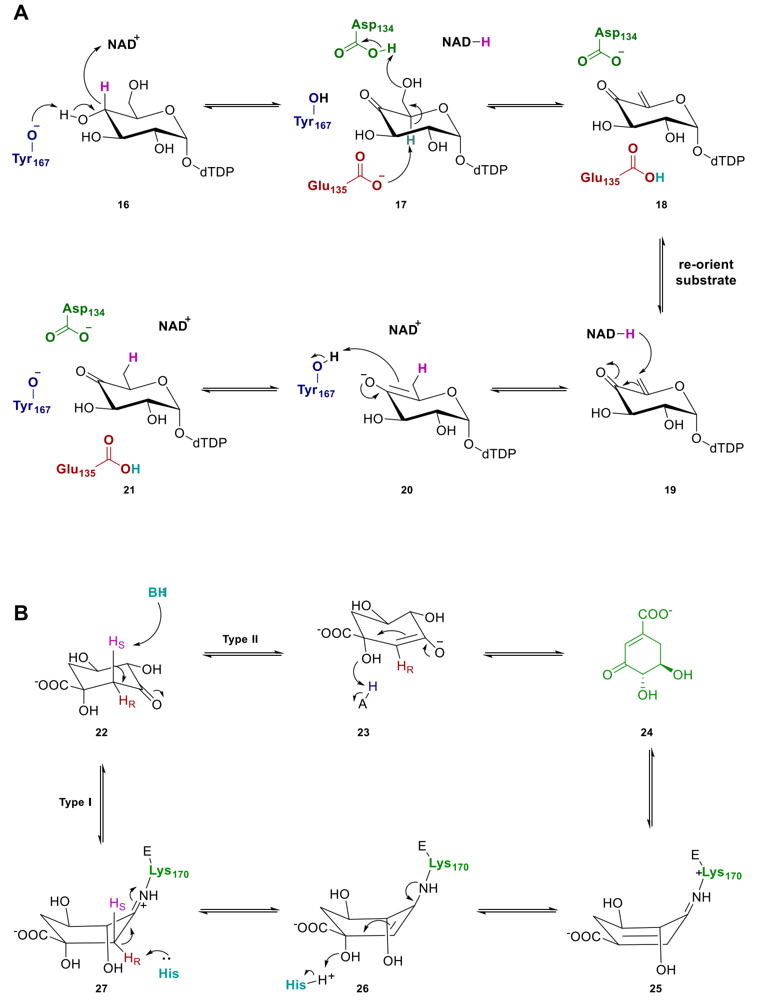

Figure S16. Dehydratase mechanisms; **A** Dehydration of dTDP-D-glucose by RmlB and NAD^{+}, in close analogy to the mechanism catalysed by TunA . **B** DHQII follows a stepwise E1cB mechanism: an active site base (Y) abstracts the HS proton of the substrate **22**, forming the enolate intermediate **23**. The elimination of water is acid-catalysed by a conserved H. DHQI goes through a multistep mechanism, including a Schiff base formation with a conserved lysine. The active site residue (H) abstracts the HR proton from intermediate 16 and subsequently protonates the hydroxyl of C1 in **27**, acting both as a general base and acid.

Bioinformatic and structural analysis of Pac13

Sequence homology comparison and alignment of the *pac13* gene product, using BLAST[11], suggested that Pac13 could belong to the cupin superfamily of proteins[12], as it contains the characteristic conserved sequences G(x)5HxH(x)3,4E(x)6G and G(x)5PxG(x)2H(x)3N. The closest homologues to Pac13 are uncharacterized hypothetical proteins from other *Streptomyces* species known to generate uridyl peptide antibiotics. For example NpsF[13], the Pac13 homologue from the very closely related napsamycin biosynthesis, shows 91/77% identity/similarity respectively to Pac13. Alignment using HHpred (Homology detection & structure prediction by HMM-HMM comparison)[14] reinforces that Pac13 has homology to both metal dependent and metal independent known cupins, including lyases and isomerases. Selected examples are GtHNL[15], a manganese-dependent hydroxynitrile lyase (PDB:4bif, E 5.70E-10), KdgF[16], a lyase involved in the metabolism of sugar uronates (PDB:5fpx, E 6.50 E-10), and FdtA[17], a dTDP-4′-Keto-6-deoxy-D-glucose-3′,4′-ketoisomerase (PDB:2pa7, E 1.80E-9). With this prediction in hand, and previous gene deletion studies on Pac13, we proposed that Pac13 is the dehydratase of the pacidamycin biosynthesis, and would be expected to belong to the cupin superfamily of proteins.

In order to study the Pac13 structure, the *pac13* gene was cloned from genomic DNA isolated from *Streptomyces coeruleorubidus* into a pET-28a – based plasmid and the protein was heterologously produced in *Escherichia coli* BL21 (DE3) cells. The enzyme was purified as an *N*-terminal-His$_8$-fusion protein, with the tag being cleaved in a later purification step. Pac13 wt crystals diffracted to 1.55 Å; however, a solution using molecular replacement was not possible due to low sequence homology with already structurally characterized enzymes in the Protein Data Bank. Structure solution therefore required the preparation of a seleno-methionine

derivative (SelMet-Pac13). This initial crystal structure verified that the enzyme was indeed a cupin. The enzyme was shown, by size exclusion chromatography, to be monomeric and a single molecule was contained within the asymmetric unit of the $P3_221$ crystal form. The jelly roll-like topology[18] of Pac13 is shown in figure 2 in the manuscript. Comparing the Pac13 coordinates with the PDB archive using the Dali server[19], allowed us to identify structurally related proteins. The first homologues (PDB: 4h7l-B, Z-score 14.8, RMSD 2.2 and PDB: 4h7l-A, Z-score 14.5, RMSD 2.1) are cupins of non-assigned function, whereas the third homologue is the lyase KdgF[16] (PDB: 5fpx, Z-score 13.4, RMSD 2.1) which catalyzes the conversion of 4,5-unsaturated digalacturonate (ΔGalUA) to 5-keto-4-deoxyuronate (DKI). The crystal structure of KdgF revealled it to be dimeric, with each monomer containing a catalytic centre comprising of a Ni^{2+}ion co-ordinated by H48, H46, H87 and Q53 (Figure S18), in stark contrast to the monomeric, metal-free Pac13.

```
Pac13     MTKYKYTVEESERFNKHGIDLTVYGQVDPSATVVRVSVERGHFQEFFNVRSSYTYYVVSG
NpsF      MTTYRHTVEDADVFSKHGIELTVYGQRDPSATVVRVQVERGHFQEFSNSRSSYIYYIVSG
SsaM      MTTYRHTVEEADRFHKHGIDLTVYGQDDPAATVVRVNVERGHFQEFLNTRSSYTYYIVSG
          **.*::***::: * ****:****** **:******.********* * **** **:***

Pac13     QGVFYLNSEAVPAGATDLITVPPNTRIHYFGSMEMVLTVAPAFNEQDERHVRFISESESPY
NpsF      RGVFHLNDEAIAVGATDLVTVPPNTRIHYFGTMEMVLTVAPAFDERDERHIRFVSESEIPD
SsaM      QGVFHLNGEPVAVGATDLITVPPNTRIFYFGAMEMVLTVAPAFDERDERHVRFISESESPG
          :***:**.* : .*****:********.***:**********:*:****:**:**** *
```

Figure S17. Comparison of Pac13 sequence with its homologues, the hypothetical proteins from the other UPAs biosynthetic clusters. Alignment was performed using Clustal-O[20].

Table S5. Alignment of Pac13 sequence using HHpred[14]; a wide range of cupins including lyases and sugar modifying enzymes; kdGF, QtdA, GthNl, YdaE, WlaRA, RemF, dTDP-4-keto-6-deoxy-D-glucose-3,4-ketoisomerases and DSMP lyases.

PDB	Enzyme	Prob	E-value	P-value	Score	SS	Cols	Querry HMM	Template HMM
5by5_A	L-ectoine synthase	99.4	2.80E-11	7.20E-16	70.4	11.6	121	1-121	1-129 (146)
1v70_A	probable antibiotics biosynthetic enzyme	99.4	7.20E-11	1.80E-15	64.1	12.2	99	1-102	1-104 (105)
3ht1_A	RemF protein	99.3	5.00E-11	1.30E-15	69.1	11.1	107	1-107	3-121 (145)
4bif_A	GtHNL	99.3	5.70E-10	1.50E-14	66	13.6	117	1-118	26-153 (156)
4e2g_A	cupin, hypothetical	99.3	9.40E-10	2.40E-14	62.1	13.4	99	1-102	14-115 (126)
5fpx_A	KdgF	99.3	6.50E-10	1.70E-14	61.4	12.2	102	1-105	6-111 (113)
1yhf_A	SPy1581, hypothetical	99.3	6.70E-10	1.70E-14	61.5	12.1	98	2-102	14-113 (115)
1vj2_A	manganese-containing cupin, hypothetical	99.2	3.10E-10	8.00E-15	64.7	10.1	102	1-102	13-123 (126)
3hqx_A	cupin, unknown	99.2	1.60E-09	4.20E-14	60.7	12.3	95	5-101	13-111 (111)
5flh_A	quercetinase	99.2	8.70E-10	2.20E-14	67.2	11.9	88	15-102	36-134
2pa7_A	FdtA, dTDP-4-keto-6-deoxy-D-glucose-3,4-ketoisomerase	99.2	1.80E-09	4.60E-14	62.3	11.7	107	1-107	2-120 (141)
2oa2_A	BH2720 protein, unknown	99.2	2.40E-10	6.30E-15	66.7	8.1	117	1-120	16-143 (148)
2gu9_A	Tetracenomycin polyketide synthesis, putative	99.2	2.30E-09	5.90E-14	59.1	11.7	88	29-116	19-113 (113)
3i7d_A	Sugar phosphate isomerase	99.1	1.80E-09	4.60E-14	64.1	10.9	102	1-102	2-122 (163)
5tpv_A	WlaRA, TDP-fucose-3,4-ketoisomerase	99.1	4.30E-09	1.10E-13	62	12.2	107	1-107	1-118 (153)
4b29_A	DSMP lyase	99.1	3.90E-09	9.90E-14	65.8	12.3	96	15-110	113-215 (217)
2pyt_A	Ethanolamine biosynthesis	99.1	3.70E-09	9.60E-14	60.4	11.1	88	16-106	42-132 (133)
4axo_A	EutQ, ethanolamine biosynthesis	99.1	3.20E-09	8.20E-14	62.4	10.6	90	15-107	50-142 (151)

5cu1_A	DSMP lyase, metal-dependent	99.1	8.70E-09	2.20E-13	63.9	12.9	100	15-115	104-210 (212)
2vpv_A	Mif2P, DNA-Binding Kinetochore Protein	99.1	9.70E-09	2.50E-13	61.3	12.4	84	15-99	73-162 (166)
2y0o_A	YdaE, probable D-lyxose ketoisomerase	99.1	5.50E-09	1.40E-13	63	10.5	88	29-116	51-169 (175)
4luk_A	Glucose-6-phosphate isomerase	99.1	1.60E-08	4.00E-13	61.6	12.6	93	15-107	47-160 (189)
4zu5_A	QtdA	99	1.50E-08	3.90E-13	58.9	11.9	107	1-107	2-120 (144)
3h7j_A	Bacilysin biosynthesis	99	6.80E-09	1.80E-13	65.2	10.9	93	29-121	143-240 (243)
2vqa_A	MncA, CucA	99	1.50E-08	3.80E-13	67.1	12.5	100	3-102	22-133 (361)

Table S6. Alignment of Pac13 coordinates with PDBeFold[21], sorted by Q-score; the list of neighbours is sorted by Z-score.

PDB entry	Enzyme	Co-factor or metal	Q	P	Z	RMSD	N_{align}	N_g	% seq	%s ee	%s ee	N_{res}
				Scoring						Query	Match	
4mv2	PLU4264, unknown function	Ni^{2+}	0.44	3.3	8.1	1.89	94	4	13	50	50	120
2f4p:B	Cupin-like protein (TM1010), unknown function	-	0.43	5.2	7.9	1.78	96	7	13	60	67	132
3cew:A	BF4112, unknown function	Zn^{2+}	0.42	2.3	7.5	1.58	87	4	14	50	56	118
1yhf:A	SPY1581, unknown function	-	0.36	1.3	6.6	2.21	88	6	13	50	50	114
2ozj:A	DSY2733, unknown function	-	0.35	5.6	8.0	2.29	86	3	14	60	75	110
2o1q:B	MPE_A3659, putative acetylacetone dioxygenase	Zn^{2+}	0.30	2.2	5.9	2.47	94	9	10	60	50	144
1cax:D	Canavalin, nutrient reservoir activity	-	0.30	3.8	6.5	2.48	106	9	14	70	64	184
2oyz:A	VPA0057, unknown function	-	0.23	0.8	4.9	2.97	72	6	6	60	60	94
2fe0:A	SMP-1 (Small Myristoylated Protein, unknown function	-	0.16	-0.0	3.2	3.45	76	9	5	50	50	131
4bd4:D	Superoxide dismutase, human, H43F	Zn^{2+}, Cu^{2+}	0.12	-0.0	1.4	3.68	62	7	8	50	63	104
1p4u:A	GGA3 GAE domain with rabaptin-5 peptide, adaptor protein	-	0.12	-0.0	2.0	4.23	79	8	13	50	50	145
4jg9:B	Putative lipoprotein	-	0.11	-0.0	1.7	5.05	83	6	5	50	50	139
5bwa:B	Ornithine Decarboxylase-PLP-Antizyme1 ternary complex	PLP	0.081	-0.0	1.5	4.58	63	7	6	50	56	121
2af9:A	GM2-Activator protein complexed with phosphatidylcholine, lipid transporter activity	-	0.068	-0.0	1.7	5.06	72	8	6	50	50	163

Table S7. Alignment of Pac13 coordinates with DALI server[19]; the list of neighbours is sorted by Z-score.

PDB	Enzyme	Z	RMSD	lali	N_{res}	%id
5fq0-A	KdgF	13.4	2.1	102	110	18
5fpz-A	Pectin degradation enzyme	13.3	2.0	102	110	15
1o4t-A	Putative oxalate decarboxylase	12.2	2.0	97	115	15
5bxx-B	L-ectoine synthase	12.2	2.2	101	113	7
2cav-A	Canavalin	12.0	2.5	112	346	12
3h50-A	Tetracenomycin polyketide syntheis	12.0	2.1	97	114	11
3ht1-A	RemF	11.9	2.9	113	141	12

4yrd-B	CapF, capsular polysaccharide synthesis	11.8	2.5	106	346	11
5u57-A	(S)-2-Hydroxtpropylphosphonic acid epoxidase	11.7	2.0	100	190	12
2y0o-A	D-lyxose ketol-isomerase	11.7	1.6	103	171	14
4o9e-B	QdtA	7.9	2.0	83	137	8
2pae-A	DTDP-6-deoxy-3,4-keto-hexulo isomerase	7.6	1.8	80	136	11

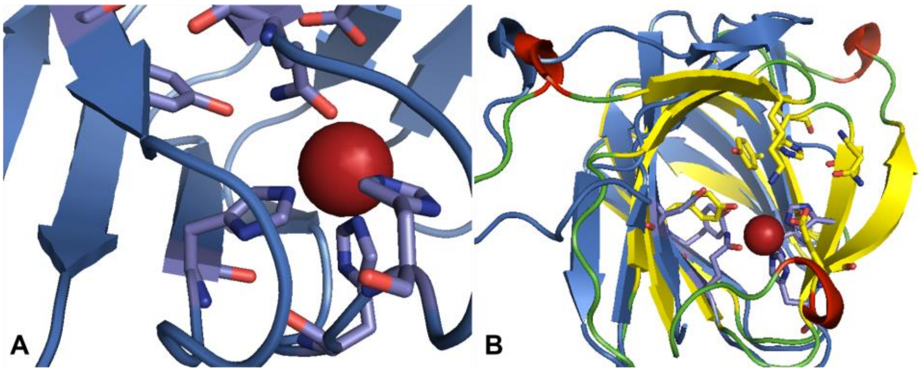

Figure S18. Metal-binding sites in KdgF and comparison with Pac13. **A**: KdgF metal binding site; H48, H46, H87, Q53 residues shown in purple and Ni^{2+} in red. **B**: Pac13 (shown in yellow) structure overlayed with KdgF (shown in blue).

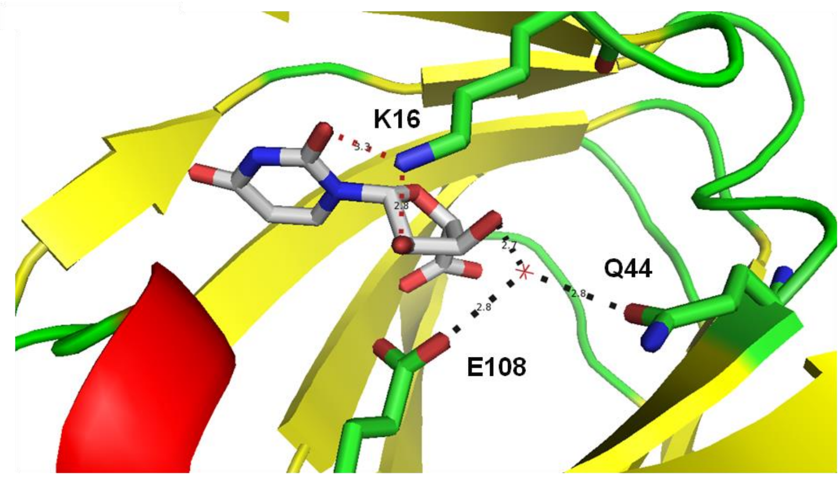

Figure S19. Active site of crystal structure of Pac13 complex with uridine 5′-uronic acid **8**. Residue E108 co-ordinates a water molecule proximal to the 3′-hydroxyl.

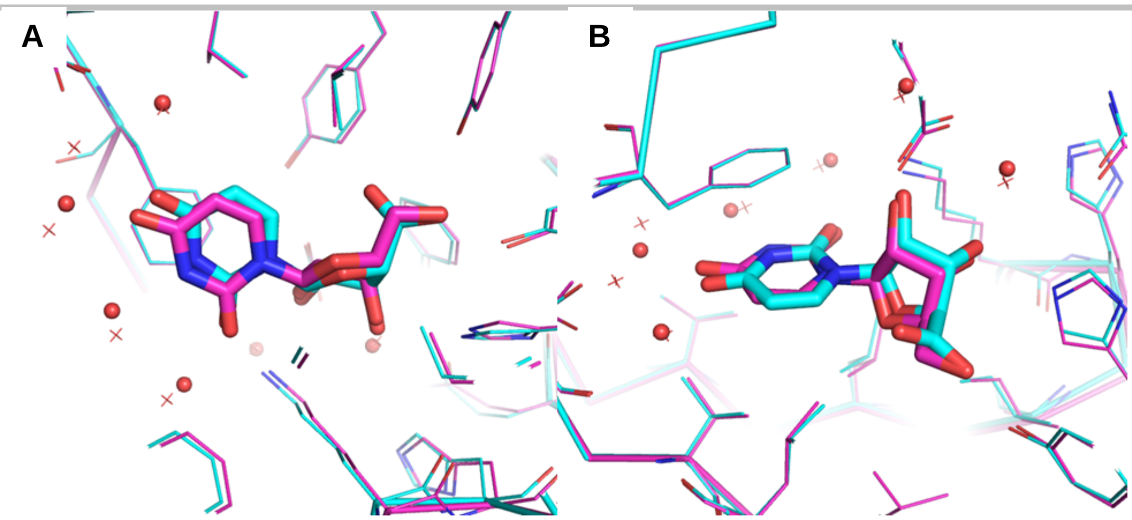

Figure S20. A, B Active site of complex of Pac13 with ligand uridine 5′-uronic acid **8** (coloured in light blue), superimposed with complex of Pac13 with uridine **7** (coloured in pink).

Aspects of the chemistry of cupins

According to the Pfam database [22] the cupin clan (CL0029), contains 61 families. The Pfam acession codes for the cupin families were used for searching solved structures in the PDB database (Table S8). After the deletion of dublicates, we have identified 361 structures, of one which only one corresponded to a dehydratase, ectoine sythase (PDB: 5BXX) [23]. Furthermore, according to our results, Pac13 remains the smallest cupin with a characterised enzymatic activity. The next entry corresponding to a characterised enzyme is the CGMP-dependent protein kinase from *Plasmodium falciparum* (PDB: 4OFF), however this PDB structure is not the complete structure of the enzyme, but just of ITS carboxyl cGMP binding domain (144 aa). Ectoine synthase (EctC) is a 33 kDa, iron-dependent cupin that catalyzes the formation of ectoine by ring closure of the substrate *N*-gamma-acetyl-$_L$-2,4-diaminobutyric acid through a water elimination reaction. Both in solution and in crystal form, EctC is a dimer with a head-to-tail arrangement.

Table S8. Selected PDB structures of cupins.

PDB	Macromolecule Name	*Source*	EC No	Classification	Structure MW	Residue Count	Ligand Name
1LKN	hypothetical protein tm1112	*Thermotoga maritima*		structural genomics, unknown function	10774.52	89	
2K9Z	uncharacterized protein TM1112	*Thermotoga maritima*		structural genomics, unknown function	10774.52	89	
2OYZ	UPF0345 protein VPA0057	*Vibrio parahaemolyticus*		STRUCTURAL GENOMICS, UNKNOWN FUNCTION	10254.11	94	
1V70	probable antibiotics synthesis protein	*Thermus thermophilus*		structural genomics, unknown function	11521.41	105	Na^+
3HQX	UPF0345 protein ACIAD0356	*Acinetobacter sp. ADP1*		structural genomics, unknown function	12451.81	111	

1YHF	hypothetical protein SPy1581	*Streptococcus pyogenes*		STRUCTURAL GENOMICS, UNKNOWN FUNCTION	12931.96	115	
2B8M	Hypothetical protein MJ0764	*Methanocaldococcus jannaschii*		UNKNOWN FUNCTION	14343.4	117	Cl^-
2MNG	Potassium/sodium hyperpolarization-activated cyclic nucleotide-gated channel 4	*Homo sapiens*		TRANSPORT PROTEIN	14985.34	131	
2D93	Rap guanine nucleotide exchange factor 6	*Homo sapiens*		SIGNALING PROTEIN	14927.87	134	
1WGP	Probable cyclic nucleotide-gated ion channel 6	*Arabidopsis thaliana*		MEMBRANE PROTEIN	15088.15	137	
2K0G	Mll3241 protein	*Mesorhizobium loti*		MEMBRANE PROTEIN	15312.56	142	ADENOSINE-3',5'-CYCLIC-MONOPHOSPHATE
2KXL	Cyclic nucleotide-gated potassium channel mll3241	*Mesorhizobium loti*		MEMBRANE PROTEIN	14983.35	142	
3MDP	Cyclic nucleotide-binding domain (CNMP-BD) protein	*Geobacter metallireducens*		Nucleotide Binding Protein	15661.34	142	1,2-ETHANEDIOL
2MHF	Uncharacterized protein	*Danio rerio*		TRANSPORT PROTEIN	16182.79	143	
4OFF	CGMP-dependent protein kinase	*Plasmodium falciparum*	* 2.7.11.12	TRANSFERASE	17338.79	144	SULFATE ION
3HT1	RemF protein	*Streptomyces resistomycificus*		LYASE	16442.27	145	Ni^{2+}
2OA2	BH2720 protein	*Bacillus halodurans*		STRUCTURAL GENOMICS, UNKNOWN FUNCTION	17187.89	148	
1E5R	PROLINE OXIDASE	*Streptomyces sp. TH1*	* 1.14.11.2 α	OXIDOREDUCTASE	67238.12	580	
5BXX	L-ectoine synthase	*Sphingopyxis alaskensis*	* 4.2.1.108	LYASE	65309.36	584	Fe^{2+}
2F4P	hypothetical protein TM1010	*Thermotoga maritima*		UNKNOWN FUNCTION	67322.2	588	1,2-ETHANEDIOL

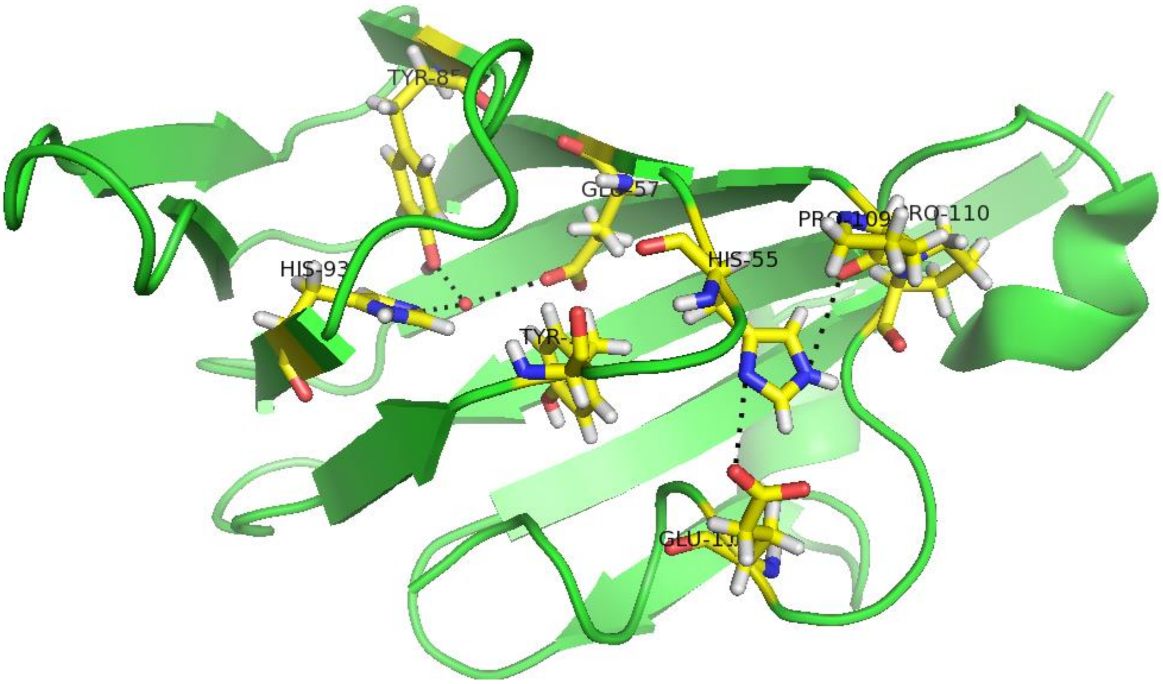

Figure S21. Proposed metal binding site in EtcC (PDB: 5BXX). Image adapted from Widderich *et al.* [23] The interactions of the water molecule (shown as red sphere) with the side chains of E57, Y85, and H93 are shown. It is suggested that the position occupied by this water molecule is probably the position of the Fe^{2+} in the active side of the enzyme. H55 interacts with the proline motif (P109 and P110) and E115.

The cupin superfamily is also classified under the entry 2.6.120.10 in the CATH database [24]. Classification of the cupin enzymes based on their enzymatc function according to CATH (Figure S22) demonstrates that only the 4.7% are lyases. Though not all cupins are metal dependent, the structurally similar to Pac13, enzymes KdgF [16] and the hydroxynitrile lyase GtHNL [15] (PDB:4bif, E 5.70E-10), rely upon nickel and manganese repsectively, in contrast to the metal-free Pac13.

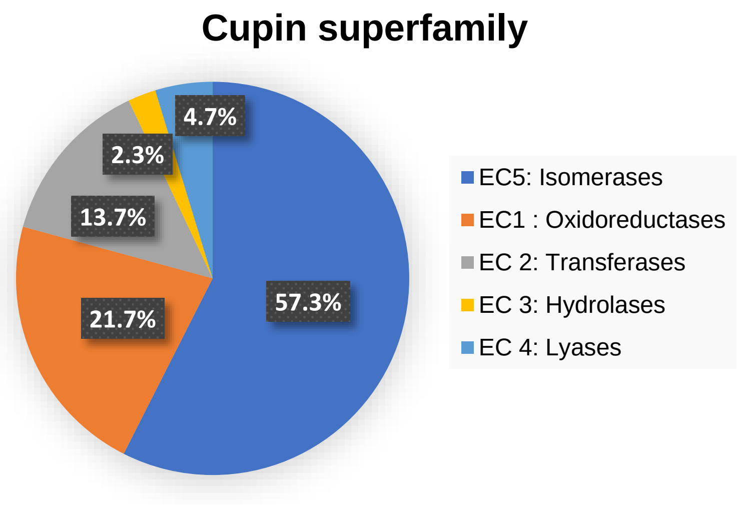

Figure S22. Enzyme classification in the cupin superfamily. Adapted from http://www.cathdb.info/version/latest/superfamily/2.60.120.10.

On the contrary, the 57.3% of enzymatic cupins are isomerases. The mechanism of isomerases that bear the cupin fold tends to be distinct of the one proposed for Pac13. The well-studied enzyme RmlC [25] (dTDP-4-dehydrorhamnose 3,5-epimerase) from the rhamnose pathway is a representative example. RmlC is a dimeric, co-factor independent cupin, that catalyses the epimerisation of the C3′ and C5′ positions of dTDP-6-deoxy-D-xylo-4-hexulose leading to dTDP-6-deoxy-L-lyxo-4-hexulose. It is proposed that an essential and conserved His acts as the active-site base, while a conserved Lys stabilizes the intermediate enolate anion and a conserved Tyr that acts as an acid. In contrast, Pac13's active site base is indeed a histidine, however, the enolate is stabilized by

the Tyr55 and Tyr89 residues and the Lys16 is clearly involved in positioning of the substrate via hydrogen bonding with the 2′ OH. Additionally, the uracil ring makes a single H-bond to Lys16 at the lip of the active site.

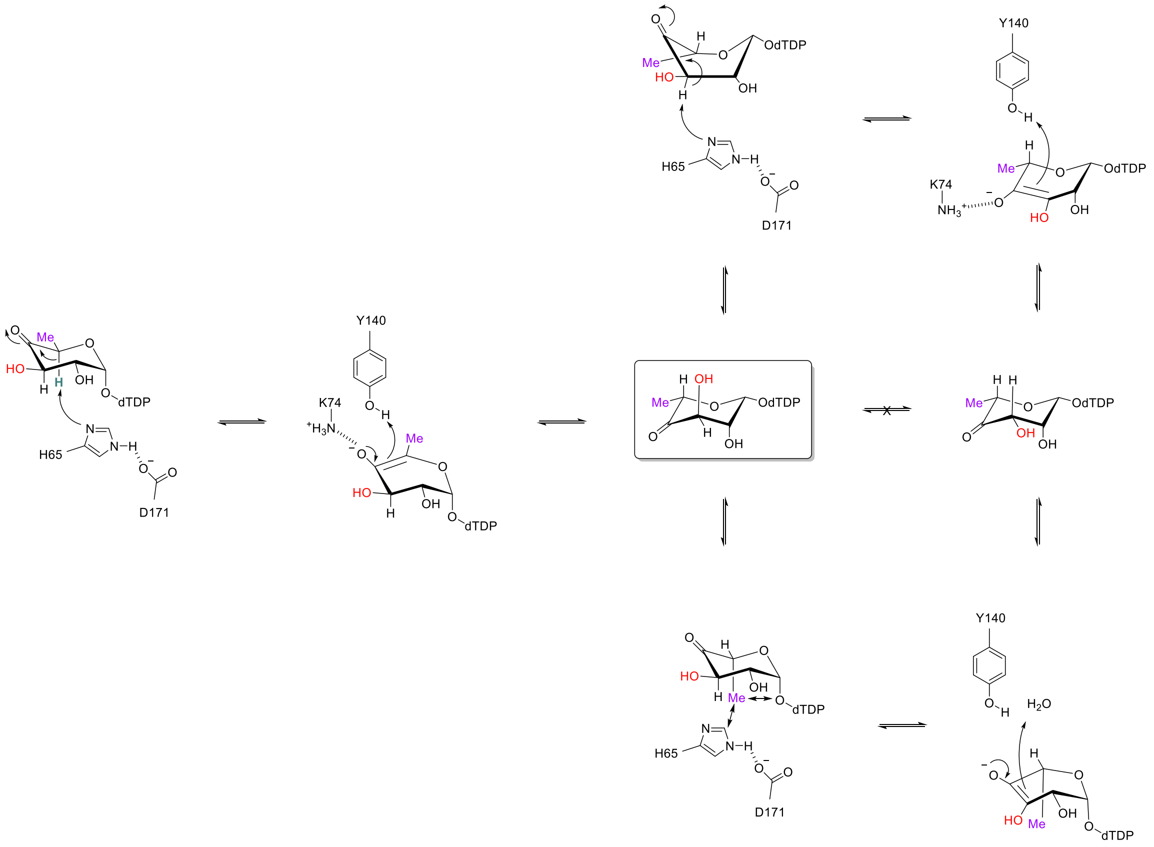

Scheme S1. Proposed mechanism of RmlC. **Adapted from Dong *et al*.**[25]

Site Directed Mutagenesis

A distinct strategy was employed in our mutant design; mutations were first modelled to ensure they conferred minimum perturbation on the steric environment of the active site; we then choose to make mutants that allowed us to address specific hypothesis as to their functional role. Residues Y89 and Y55 were mutated independently to a phenylalanine, rather than alanine, as this ′deoxy′ tyrosine analogue, would enable us to directly examine if the hydroxyl groups are involved in catalysis as speculated. H42 was mutated independently to both Q and E; we proposed that H42Q would be inactive, as a glutamine would not be able to function either as an acid or a base, whereas a glutamate could be expected to perform both as an acid and base although with lower pKa than reported in the literature [7, 22]. K16 was mutated to an R, and E108 to a Q, as we aimed to probe that the above amino acids served mainly to targeting the correct orientation of the substrate, rather being required for actual catalysis; hence, by maintaining the size and steric hindrance, we would expect the differences in the electronic environment not to be detrimental for enzyme activity.

The H42E mutant was found impossible to produce, indicating the importance of this residue for the integrity of the protein. The kinetic measurements for the activity of Y55F and K16R demonstrated lower affinity and catalytic efficiency than wt Pac13 (Table 1 MS). Mutation of K16 to an R did not abolish activity, demonstrating that K16 is not an essential lysine involved in a Schiff-base formation; hence a DHQ I – type mechanism could be further excluded with confidence. The kinetic constants for Y89F and E108Q could not be determined under initial velocity conditions employed for wt, K16R and Y55F due to their considerably lower activity. However, when all the mutants and wt enzyme were assayed under a fixed substrate and enzyme concentration, a comprehensive comparison could be derived (Table 1 MS).

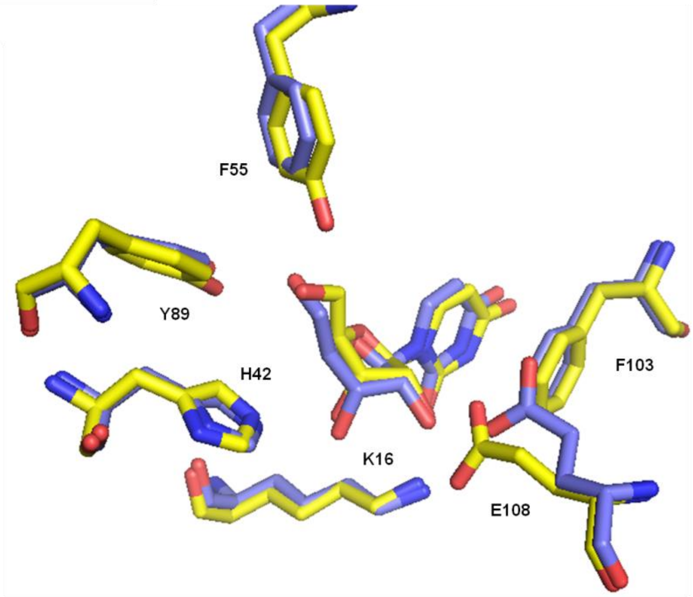

Figure S23. Complex of Y55F with uridine **7** (colored in blue, PDB:5OO9) superimposed with Pac13 wt complex with uridine **7** (colored in yellow).

pH profile analysis of Pac13 and mechanistic information derived from NMR assays at two different pH values

If the initial formation of the enolate is rapidly reversible compared to the subsequent elimination we would expect to see exchange of the 4′ proton with deuterium from the D_2O solvent. However, the ^1H NMR data at different time points of the assay provided no evidence for this demonstrating that the rate of depletion of the 4′ proton of the substrate is faster than the rate of depletion of the protons H5 or H6 of the uracil ring, indicating a high commitment to catalysis such that elimination of water appears not to be the rate limiting step. In an attempt to perturb the relative rates, we also looked for wash out of the 4′ proton at pH 9, under which conditions potentially deprotonation would be accelerated relative to elimination, but no evidence for exchange was obtained.

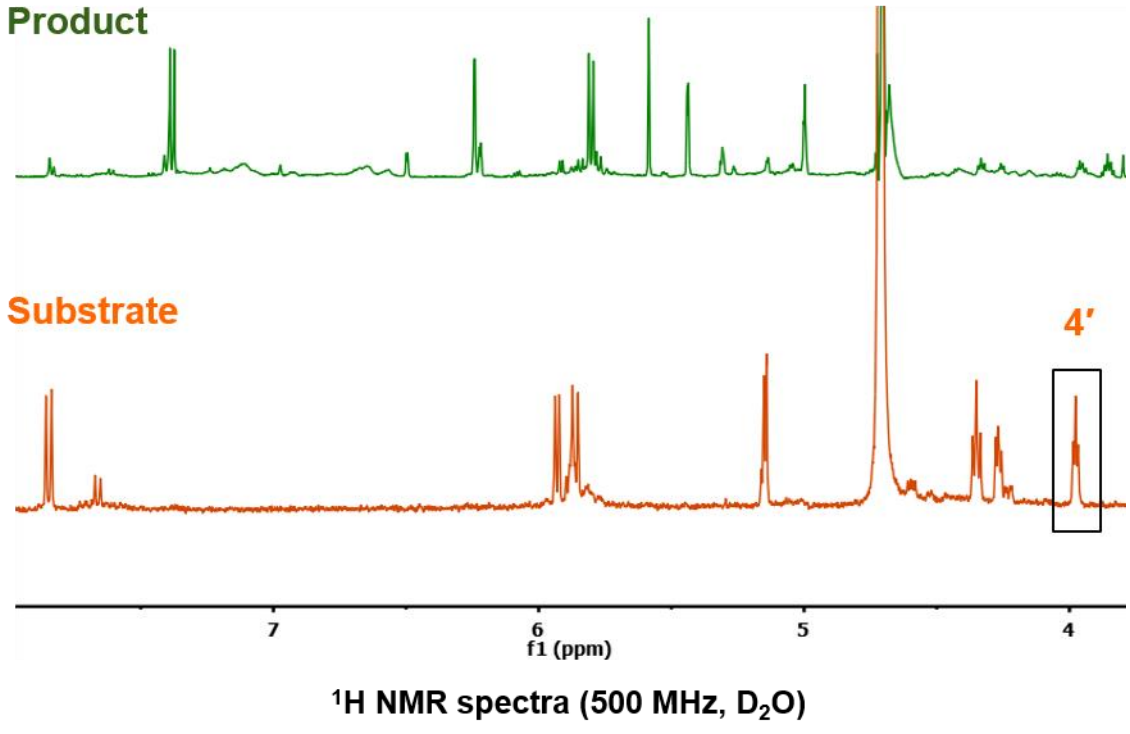

Figure S24: ^1H NMR spectra of the first assay timepoint, demonstrating the substrate (uridine -5′-aldehyde **1**, in orange colour) and at later timepoint, demonstrating product (3′-deoxy-3′,4′-didehydrouridine-5′-aldehyde **2**, in green colour). The 4′ proton of substrate 1 that can be used for reaction monitoring, is shown.

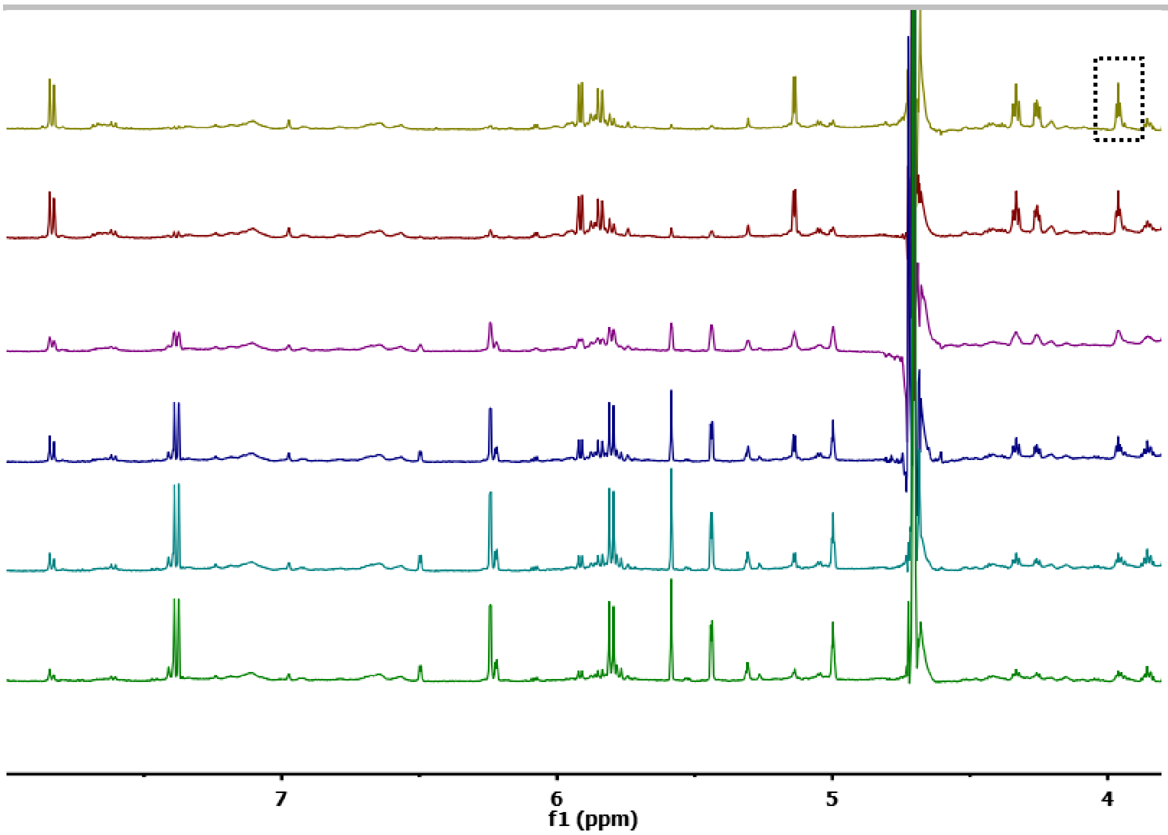

Figure S25: ^{1}H NMR (D_2O, 500 MHz) assay, monitoring the reaction of Pac13 with uridine-5′-aldehyde **1** and the formation of 3′-deoxy-3′,4′-didehydrouridine-5′-aldehyde **2** at pH 7.5.

The pH profile analysis (Figure S13) demonstrated that the enzyme was completely inactive below pH 5.4 and above pH 10. The profile followed a bell-shaped curve, with pKa_1 and pKa_2 values of 6.2 and 8.6 respectively. Since the substrate does not have any ionisable groups in this pH range, this result supports our proposal of a histidine acting as a general base to abstract the 4′ proton[23]. This is consistent with previous pKa values for histidines reported in the literature (6.3 ± 0.1 for H40 and H57 of the colicin E7 protein[24] and 6.72 ± 0.02, 6.19 ± 0.04 and 5.79 ± 0.07 for H105, H119, H12 of ribonuclease A respectively)[23]. The upper pKa may be postulated as due to Y89 or Y55 needing to be protonated so as to polarize the aldehyde and is in accordance with the apparent pKa of Y48 (8.4 ± 0.1)[25] from human aldolase reductase, a tyrosine residue that has been demonstrated to act as a proton donor. Despite such pKa values being lower than the intrinsic pKa for a tyrosine residue in active sites (pKa of 9.7)[26] or for a free tyrosine hydroxyl group in solution (pKa of 10.5)[25], perturbed pKa values for tyrosines in enzyme active sites have been previously reported[26-27]. However, the involvement of ionisable reaction intermediates such as the proposed enolate and their impact on the observed pKas cannot be excluded[28]. Furthermore, there is no obvious residue positioned to protonate the leaving hydroxide ion and this role may be fulfilled by H42 or by a solvent molecule mediated through E108 to eventual His deprotonation. Though unusual, a histidine residue mediating as a general base and acid in two sequential steps, has been reported for enzymes in the literature, such as the dehydratases DHQ I[8b] from *Salmonella typhi* and QmnD4[29] from *Amycolatopsis orientalis*, the quartromicin producer, as well as the well-studied enzymes ribonuclease A and triose phosphate isomerase[30].

References

[1] a) X. Chi, P. Pahari, K. Nonaka, S. G. Van Lanen, *J Am Chem Soc* **2011**, *133*, 14452-14459; b) A. E. Ragab, S. Grüschow, D. R. Tromans, R. J. M. Goss, *J Am Chem Soc* **2011**, *133*, 15288-15291; c) Gordon H. Jones, Masao Taniguchi, Derek Tegg, J. G. Moffatt, *J. Org. Chem.* **1978**, *44*, 1979-1309.

[2] a) M. Petrova, M. Budesınsky, E. Zbornıkova, P. Fiedler, I. Rosenberg, *Org Lett* **2011**, *13*, 4200-4203. b) M. Petrova, M. Budesınsky, I. Rosenberg, *Tetrahedron Lett.* **2010**, *51*, 6874-6876.

[3] B. S. Chen, L. G. Otten, U. Hanefeld, *Biotechnol Adv* **2015**, *33*, 526-546.
[4] a) W. Buckel, J. Zhang, P. Friedrich, A. Parthasarathy, H. Li, I. Djurdjevic, H. Dobbek, B. M. Martins, *Biochim Biophys Acta* **2012**, *1824*, 1278-1290; b) T. Toraya, *Arch Biochem Biophys* **2014**, *544*, 40-57.
[5] a) M.-F. Giraud, J. H. Naismith, *Current Opinion in Structural Biology* **2000**, *10*, 687-696; b) R. A. Field, J. H. Naismith, *Biochemistry* **2003**, *42*, 7637-7647; c) S. T. M. Allard, K. Beis, M.-F. Giraud, J. W. Gross, C. Whitfield, P. Messner, D. J. Maskell, A. D. Hegeman, R. C. Wilmouth, M. Graninger, A. G. Allen, J. H. Naismith, *Structure* **2002**, *10*, 81-92.
[6] N. A. Webb, A. M. Mulichak, J. S. Lam, H. L. Rocchetta, R. M. Garavito, *Protein Sci* **2004**, *13*, 529-539.
[7] F. J. Wyszynski, S. S. LeFe, T. Yabe, H. Wang, J. P. Gomez-Escribano, M. J. Bibb, S. J. Lee, G. J. Davies, B. G. Davis, *Nat Chem* **2012**, *4*, 539-546.
[8] a) C. Coderch, E. Lence, A. Peon, H. Lamb, A. R. Hawkins, F. Gago, C. Gonzalez-Bello, *Biochem J* **2014**, *458*, 547-557; b) S. H. Light, G. Minasov, L. Shuvalova, M. E. Duban, M. Caffrey, W. F. Anderson, A. Lavie, *J Biol Chem* **2011**, *286*, 3531-3539; c) C. Gonzalez-Bello, L. Tizon, E. Lence, J. M. Otero, M. J. van Raaij, M. Martinez-Guitian, A. Beceiro, P. Thompson, A. R. Hawkins, *J Am Chem Soc* **2015**, *137*, 9333-9343.
[9] A. W. Roszak, T. Krell, M. Fredrickson, J. R. Coggins, D. A. Robinson, I. S. Hunter, C. Abell, A. J. Lapthorn, *Structure* **2002**, *10*, 493-503.
[10] B. M. Nestl, C. Geinitz, S. Popa, S. Rizek, R. J. Haselbeck, R. Stephen, M. A. Noble, M.-P. Fischer, E. C. Ralph, H. T. Hau, H. Man, M. Omar, J. P. Turkenburg, S. van Dien, S. J. Culler, G. Grogan, B. Hauer, *Nature Chemical Biology* **2017**.
[11] G. M. Boratyn, C. Camacho, P. S. Cooper, G. Coulouris, A. Fong, N. Ma, T. L. Madden, W. T. Matten, S. D. McGinnis, Y. Merezhuk, Y. Raytselis, E. W. Sayers, T. Tao, J. Ye, I. Zaretskaya, *Nucleic Acids Res* **2013**, *41*, 29-33.
[12] a) S. Khuri, F. T. Bakker, J. M. Dunwell, *Mol. Biol. Evol.* **2001**, *18*, 593-605; b) J. M. Dunwell, A. Purvis, S. Khuri, *Phytochemistry* **2004**, *65*, 7-17.
[13] L. Kaysser, X. Tang, E. Wemakor, K. Sedding, S. Hennig, S. Siebenberg, B. Gust, *Chembiochem* **2011**, *12*, 477-487.
[14] J. Soding, A. Biegert, A. N. Lupas, *Nucleic Acids Res* **2005**, *33*, 244-248.
[15] I. Hajnal, A. Lyskowski, U. Hanefeld, K. Gruber, H. Schwab, K. Steiner, *FEBS J* **2013**, *280*, 5815-5828.
[16] J. K. Hobbsa, S. M. Leea, M. Robba, F. Hofb, C. Barrb, K. T. Abea, J.-H. Hehemanna, R. McLeane, D. W. Abbotte, A. B. Borastona, *Proc Natl Acad Sci U S A* **2016**, *113*, 6188–6193
[17] a) M. L. Davis, J. B. Thoden, H. M. Holden, *J Biol Chem* **2007**, *282*, 19227-19236; b) J. B. Thoden, E. Vinogradov, M. Gilbert, A. J. Salinger, H. M. Holden, *Biochemistry* **2015**, *54*, 4495-4506.
[18] a) W. Aik, M. A. McDonough, A. Thalhammer, R. Chowdhury, C. J. Schofield, *Curr Opin Struct Biol* **2012**, *22*, 691-700; b) J. M. Dunwell, *Biotechnology and Genetic Engineering Reviews* **1998**, *15*, 2-32.
[19] L. Holm, P. Rosenstrom, *Nucleic Acids Research* **2010**, *38*, 545-549.
[20] M. Goujon, H. McWilliam, W. Li, F. Valentin, S. Squizzato, J. Paern, R. Lopez, *Nucleic Acids Res* **2010**, *38*, 695-699.
[21] E. Krissinel, K. Henrick, *Acta Crystallographica* **2004**, *60*, 2256-2268.
[22] R. D. Finn, A. Bateman, J. Clements, P. Coggill, R. Y. Eberhardt, S. R. Eddy, A. Heger, K. Hetherington, L. Holm, J. Mistry, E. L. L. Sonnhammer, J. Tate, M. Punta, *Nucleic Acids Res.* **2014**, *42*, 222–230.
[23] N. Widderich, S. Kobus, A. Höppner, R. Riclea, A. Seubert, *PLOS ONE* **2016**, *11*, e0151285.
[24] C. A. Orengo, A. D. Michie, S. Jones, D. T. Jones, M. B. Swindells, J. M. Thornton, *Structure* **1997**, *5*, 1093-1109
[25] C. Dong, L. L. Major, V. Srikannathasan, J. C. Errey, M.-F. Giraud, J. S. Lam, M.Graninger, P. Messner, M. R. McNeil, R. A. Field, C. Whitfield, J. H. Naismith, *J. Mol. Biol.* **2007**, *365*, 146–159
[26] A. Fersht, The pH Dependence of Enzyme Catalysis. In *Structure And Mechanism in Protein Science;* W. H. Freeman & Company: New York, **1997**; 169-190.
[27] J. L. Markley, *Biochemistry* **1975**, *14*, 3546-3553.
[28] A. L. Hansen, L. E. Kay, *Proc Natl Acad Sci U S A* **2014**, *111*, 1705-1712.
[29] K. M. Bohren, C. E. Grimshaw, C.-J. Lai, D. H. Harrison, D. Ring, G. A. Petsko, K. H. Gabbay, *Biochemistry* **1994**, *33*, 2021-2032.
[30] T. K. Harris, G. J. Turner, *IUBMB Life* **2002**, *53*, 85-98.
[31] J. Lamotte-Brasseur, A. Dubus, R. C. Wade, *PROTEINS: Structure, Function, and Genetics* **2000**, *40*, 23-28.
[32] B. Plaut, J. R. Knowles, *Biochem. J.* **1972**, *129*, 311-320.
[33] L. F. Wu, H. Y. He, H. X. Pan, L. Han, R. Wang, G. L. Tang, *Org Lett* **2014**, *16*, 1578-1581.
[34] D. Withford, Enzyme kinetics, structure, function, and catalysis. In *Proteins, Structure and Function*; John Wiley & Sons, Ltd: Chichester, West Sussex, England, **2005**, 214-218.
